# Entropy Generation and Thermal Radiation Analysis of EMHD Jeffrey Nanofluid Flow: Applications in Solar Energy

**DOI:** 10.3390/nano13030544

**Published:** 2023-01-29

**Authors:** Bhupendra Kumar Sharma, Anup Kumar, Rishu Gandhi, Muhammad Mubashir Bhatti, Nidhish Kumar Mishra

**Affiliations:** 1 Department of Mathematics, Birla Institute of Technology and Science, Pilani 333031, India; 2 College of Mathematics and Systems Science, Shandong University of Science and Technology, Qingdao 266590, China; 3 Material Science, Innovation and Modelling (MaSIM) Research Focus Area, North-West University, Mafikeng Campus, Private Bag X2046, Mmabatho 2735, South Africa; 4 Department of Basic Science, College of Science and Theoretical Studies, Saudi Electronic University, Riyadh 11673, Saudi Arabia

**Keywords:** solar radiations, exponential heat source, copper nanoparticles, gyrotactic motile microorganisms, EMHD, Joule heating

## Abstract

This article examines the effects of entropy generation, heat transmission, and mass transfer on the flow of Jeffrey fluid under the influence of solar radiation in the presence of copper nanoparticles and gyrotactic microorganisms, with polyvinyl alcohol–water serving as the base fluid. The impact of source terms such as Joule heating, viscous dissipation, and the exponential heat source is analyzed via a nonlinear elongating surface of nonuniform thickness. The development of an efficient numerical model describing the flow and thermal characteristics of a parabolic trough solar collector (PTSC) installed on a solar plate is underway as the use of solar plates in various devices continues to increase. Governing PDEs are first converted into ODEs using a suitable similarity transformation. The resulting higher-order coupled ODEs are converted into a system of first-order ODEs and then solved using the RK 4th-order method with shooting technique. The remarkable impacts of pertinent parameters such as Deborah number, magnetic field parameter, electric field parameter, Grashof number, solutal Grashof number, Prandtl number, Eckert number, exponential heat source parameter, Lewis number, chemical reaction parameter, bioconvection Lewis number, and Peclet number associated with the flow properties are discussed graphically. The increase in the radiation parameter and volume fraction of the nanoparticles enhances the temperature profile. The Bejan number and entropy generation rate increase with the rise in diffusion parameter and bioconvection diffusion parameter. The novelty of the present work is analyzing the entropy generation and solar radiation effects in the presence of motile gyrotactic microorganisms and copper nanoparticles with polyvinyl alcohol–water as the base fluid under the influence of the source terms, such as viscous dissipation, Ohmic heating, exponential heat source, and chemical reaction of the electromagnetohydrodynamic (EMHD) Jeffrey fluid flow. The non-Newtonian nanofluids have proven their great potential for heat transfer processes, which have various applications in cooling microchips, solar energy systems, and thermal energy technologies.

## 1. Introduction

Solar energy is one of the most significant renewable energy sources with negligible adverse environmental effects. This technology provides clean and inexhaustible energy without burning any fuel. The use of solar energy is directly connected to the natural resources of heat, water, and electricity. It is presently a difficult issue in the field of solar energy storage to improve the thermal efficiency of solar collectors to meet the demands and needs of energy in industrial and technical applications. Solar collector performance and operation are also impacted by inadequate heat transmission and thermophysical properties of the base fluid. Numerous efforts have been made in this direction to improve the thermophysical properties of the base fluids. Nanofluids are the next-generation fluids that not only discover surprising thermal features but also result in high thermal properties. Adding nanoparticles to fluids can enhance heat transmission and solar energy storage. Mushtaq et al. [1] investigated the impact of solar radiation on the 2D stagnation-point flow of a nanofluid. The MHD 3D Jeffrey fluid flow characteristics for heat transfer in solar energy applications were analyzed by Shehzad et al. [2]. The 3D nanofluid flow of the boundary layer for solar energy applications was investigated by Khan et al. in [3,4]. The effect of silver and copper nanoparticles on the unsteady MHD Jeffrey fluid flow was described by Zin et al. [5]. Reddy et al. [6] reviewed the applications of nanofluids in solar conversion systems and performance enhancement. Daniel et al. [7] described the impact of thermal radiation on the flow of EMHD nanofluid over a nonuniformly thick stretching sheet. The effects of thermal radiation on the EMHD nanofluid flow over a stretching sheet with nonuniform thickness was described by Daniel et al. [7]. Wahab et al. [8] discussed the potential of nanofluids in the efficiencies of solar collectors. Numerical simulation for the effects of solar energy on the turbulence of MHD flow of cross nanofluid was explored by Azam et al. [9]. The radiation effects on the peristaltic flow of gold nanoparticles with double-diffusive convection across an asymmetric channel were discussed by Sunitha et al. [10]. The thermal behavior of a hybrid nanofluid flow in the presence of radiative solar energy inside a microchannel was studied by Acharya [11]. The significance of copper and alumina nanoparticles migrating across the water with a convectively heated surface was analyzed by Song et al. [12]. Jamshed et al. examined the heat transmission in Maxwell nanofluid flow across an exponentially uniform stretchable plate within the parabolic trough solar collector. The optimization of solar energy using Sutterby hybrid nanofluid in solar HVAC subjected to expanding sheets was also addressed by Jamshed et al. [13]. Recently, some research ([14,15,16,17]) has examined the effect of nanoparticles in different types of fluids. A comparison of thermal characteristics of Cu and Ag nanoparticle suspensions in sodium alginate for Sutterby nanofluid flow in solar collectors was conducted by Bouslimi et al. [18]. Shahzad et al. [19] investigated the thermal cooling efficiency of a solar water pump by using Oldroyd-B (aluminum alloy–titanium alloy/engine oil) hybrid nanofluid.

Thermophoresis is the force exerted by particles suspended in a fluid in response to the heat gradient, also known as thermophoresis or thermal diffusion. The random motion of particles suspended in the liquid results in collisions when it collides with another particle. Furthermore, these collisions of particles cause a random or zigzag motion. It involves the transfer of energy between the particles, and this phenomenon is known as Brownian diffusion. Pakravan and Yaghoubi [20] explored the heat transfer with Dufour and the thermophoresis effect of nanoparticles. Anbuchezhian et al. [21] studied heat transfer corresponding to solar radiations under the effects of the thermophoresis and Brownian motion. Kandasamy et al. [22] presented the effect of thermophoresis and Brownian motion under temperature stratification owing to sun radiation on nanofluid flow. Hayat et al. [23] examined the heat transfer with thermophoretic diffusion and Brownian motion through a stretching cylinder of the Jeffrey nanofluid flow. The effects of magnetohydrodynamics, thermophoresis, and Brownian motion on the radiative nanofluid flow across a rotating sheet were examined by Mabood et al. [24]. Sulochana et al. [25] reviewed the impact of transpiration on a Carreau nanofluid flow with Brownian motion and thermophoresis diffusion via a stretching sheet. Applications of solar radiation under the cumulative effects of thermophoresis and Brownian motion for CuO–Water nanofluid by natural convection were investigated by Astanina et al. [26]. Awan et al. [27] analyzed the radiative heat transfer in MHD nanofluid flow with the effects of thermophoresis, Brownian motion, and solar energy. Rekha et al. [28] investigated the influence of thermophoretic particle deposition under solar radiation impacts on heat transfer for nanofluid flow in various geometries. Recently, some research has been conducted to study the thermophoresis and Brownian motion impacts under different types of considerations (in the articles [29,30,31]) past vertical and horizontal surfaces.

Entropy is a system’s thermal energy which is unavailable for any valuable work. Entropy generation generally measures the performance of engineering systems. Entropy production improves a system’s performance and destroys significant amounts of energy. Studying the entropy generation in solar collectors is essential to optimize the amount of energy accessible. Lowering the entropy generation rate in constructing practical systems is preferable. The effect of direct absorption solar collectors for heat transfer and entropy generation was studied by Parvin et al. [32]. The efficiency of entropy formation in the viscous fluid flow past a porous rotating disk was explored by Khan et al. [33] by using the theory for the partial slip under nonlinear radiation effect. Analysis of entropy generation of a two-phase thermosyphon for solar collectors was explored by Wang et al. [34]. Several types of research ([35,36,37]) have been conducted to examine the influence of entropy generation in the existence of moving gyrotactic microorganisms with different considerations. The dynamics of mixed Marangoni convective flow for entropy generation using aluminum oxide and copper nanofluids in solar energy storage was analyzed by Li et al. [38]. Recently, Sharma et al. [39] discussed the entropy optimization in MHD thermally radiating flow with hybrid nanoparticles through a tapered multistenosed artery. Khanduri and Sharma [40] analyzed the entropy generation assuming the viscosity and thermal conductivity of the fluid to be temperature-dependent. Entropy generation for nonlinear radiation and thermodynamic behavior of a hybridized Prandtl–Eyring magneto-nanofluid for a solar aircraft were studied by Salawu et al. [41].

The gyrotactic motile microorganism is one of the particular microorganisms which moves due to the torque caused by viscous and gravity forces present in the system. The upswing suspension of gyrotactic microorganisms in the bioconvection mechanism will enhance nanoparticle stability. The system’s stability to support the suspension of nanoparticles with moving gyrotactic microorganisms was discussed by Avramenko and Kuznetsov [42]. Later, Avramenko and Kuznetsov [43] also investigated the thermal stability in the bioconvection of gyrotactic microorganisms with a primary vertical temperature gradient. Mutuku and Makinde [44] examined the bioconvection driven by a new type of water-based nanofluid that is hydromagnetically flowing across a porous vertical moving surface suspended with nanoparticles. The impact of solar radiation on gyrotactic microorganisms’ bioconvection nano-fluid flow was explored by Acharya et al. [45]. The behavior of the nanoparticles and microorganisms on the MHD Jeffrey nanofluid flow due to a rotating vertical cone was described by Saleem et al. [46]. The entropy of Maxwell nanofluid considering the existence of motile microorganisms with heterogeneous–homogeneous reactions was explored by Sohail et al. [47]. Song et al. [48] studied the mixed bioconvection flow of nanofluid with varying Prandtl numbers across a vertically moving thin needle for the aspects of solar energy. MHD Jeffrey nanofluid flow past a vertical stretching sheet under the effects of thermal partial and radiation slip with motile microorganisms was investigated by Naidu et al. [49]. Gyrotactic microorganisms in MHD flow through porous material via an inclined elongating sheet to examine heat transfer was carried out by Sharma et al. [50]. Bhatti et al. [51] explored the gyrotactic microorganisms swimming between rotating circular plates embedded in porous medium for thermal energy storage. Gyrotactic microorganisms swimming under the solar biomimetic system above the esophagus for hyperbolic tangent blood nanomaterial were examined by Hussain and Farooq [52].

Many industrial operations, including electricity production, heating or cooling processes, chemical processes, and microelectronics, depend on common fluids, including water, ethylene glycol, and heat transfer oil. In thermal engineering devices, these fluids cannot attain effective heat transfer rates due to their relatively low thermal conductivity. Utilizing ultrafine solid particles suspended in ordinary fluids to boost their heat conductivity is one method for overcoming this barrier. From the literature [53], it is noted that polyvinyl alcohol–water-based fluid with copper nanoparticles has higher temperature profiles and heat transfer rates. The previous literature is limited to the entropy generation of the Jeffrey nanofluid flows in heat and mass transfer processes over the stretching surfaces of uniform thickness. No attempts have been made to analyze the entropy generation of the EMHD Jeffrey fluid flow with motile gyrotactic microorganisms via vertical surfaces stretching nonlinearly with nonuniform thickness under the influence of solar radiations. Therefore, this study investigates the entropy generation and solar radiation effects in the presence of motile gyrotactic microorganisms and copper nanoparticles with polyvinyl alcohol–water as the base fluid. The influence of the source terms such as viscous dissipation, Ohmic heating, exponential heat source, and chemical reaction of the EMHD Jeffrey fluid flow past a vertical nonlinearly elongating surface of nonuniform thickness was studied.

## 2. Formulation of Model

### 2.1. Physical Assumptions

Consider time-independent, laminar, 2D, incompressible, non-Newtonian Jeffrey nanofluid flow with motile gyrotactic microorganisms incorporating Copper nanoparticles in the polyvinyl alcohol–water base fluid. The flow is subjected to a nonlinear elongating surface with nonuniform thickness under Joule heating, exponential heat source, viscous dissipation, and chemical reaction. It is also assumed that the motile microorganisms swimming direction and speed are unaffected by the suspended nanoparticles. The $x_{1}^{*}$ and $y_{1}^{*}$ are the axes along the vertical and horizontal direction, having corresponding components of velocities as $u_{1}^{*}$ and $v_{1}^{*}$, respectively. The influence of the induced magnetic field is minimal and is therefore neglected by assuming a very small magnetic Reynolds number (Re<<1).

**Figure 1:**
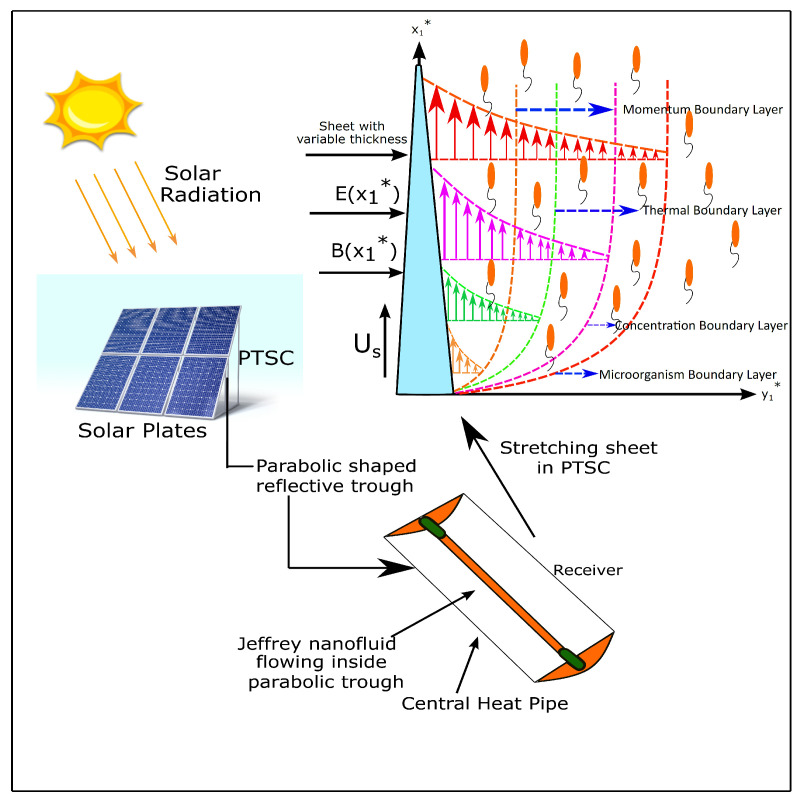
An illustration of the mathematical model.

The current density formulated by Ohm’s law is **J=σ(E+V×B)**, where **E** is the strength of the applied electric field, **V** refers the fluid velocity, and **B** is the strength of the applied magnetic field. In fluid flows of high electrical conductivity, flow control can be made in the absence of any external electric field. For that case, the volume density induced due to the Lorentz force is formulated as **F=J×B**. Therefore, in this case, the moderate applied magnetic field will induce a sufficiently strong electrical current density without any external electric field. Hence, the expression for the electrical current density and Lorentz force becomes **J=σ(V×B)** and **F=σ(V×B)×B**. However, in the case of low electrical conductivity of fluids, the current density in the absence of an electric field is minimal to achieve the flow control, even for the magnetic field of several Teslas. Therefore, an external electric field is needed for efficient flow control. The electrical current density in this case is formulated as **J=σ(E+V×B)**. Figure 1 illustrates the mathematical model.

The stress tensor components for the Jeffrey fluid model are given by

(1) $$\begin{array}{c} T=-PI+S, \end{array}$$

where ***S*** in mathematical form is expressed as

(2) $$\begin{array}{c} S=\frac{\mu_{nf}}{1+\lambda_{1}}\left[A+\lambda_{2}\frac{dA}{dt}\right], \end{array}$$

***A*** is the first-order Rivlin Erection tensor given by

$$A=\nabla V+{\left(\nabla V\right)}^{\prime }$$

where ${}^{\prime }$ denotes the transpose. 

### 2.2. Governing Equations of the Physical Model

The governing equations for Jeffrey nanofluid flow derived using the prior assumptions, the order of magnitude approach, and the typical Boussinesq approximation for the boundary layer are ([54])

(3) $$\frac{\partial u_{1}^{*}}{\partial x_{1}^{*}}+\frac{\partial v_{1}^{*}}{\partial y_{1}^{*}}=0,$$

(4) $$\begin{array}{c} u_{1}^{*}\frac{\partial u_{1}^{*}}{\partial x_{1}^{*}}+v_{1}^{*}\frac{\partial u_{1}^{*}}{\partial x_{1}^{*}}=\frac{\nu_{nf}}{\left(1+\lambda_{1}\right)}[\frac{\partial^{2}u_{1}^{*}}{\partial y_{1}^{*2}}+\lambda_{2}(u_{1}^{*}\frac{\partial^{3}u_{1}^{*}}{\partial x_{1}^{*}\partial y_{1}^{*2}}+\frac{\partial u_{1}^{*}}{\partial y_{1}^{*}}\frac{\partial^{2}u_{1}^{*}}{\partial x_{1}^{*}\partial y_{1}^{*}}+v_{1}^{*}\frac{\partial^{3}u_{1}^{*}}{\partial y_{1}^{*3}}\\ +\frac{\partial v_{1}^{*}}{\partial y_{1}^{*}}\frac{\partial^{2}u_{1}^{*}}{\partial y_{1}^{*2}})]-\frac{\sigma_{nf}}{\rho_{nf}}\left(B^{2}u_{1}^{*}-EB\right)+g{\left(\beta_{T}\right)}_{nf}\left(T_{1}^{*}-T_{\infty }^{*}\right)+g{\left(\beta_{C}\right)}_{nf}\left(C_{1}^{*}-C_{\infty }^{*}\right)\\ -g{\left(\beta_{N}\right)}_{nf}\left(N_{1}^{*}-N_{\infty }^{*}\right), \end{array}$$

(5) $$\begin{array}{c} u_{1}^{*}\frac{\partial T_{1}^{*}}{\partial x_{1}^{*}}+v_{1}^{*}\frac{\partial T_{1}^{*}}{\partial y_{1}^{*}}=\frac{\kappa_{nf}}{{\left(\rho Cp\right)}_{nf}}\frac{\partial^{2}T_{1}^{*}}{\partial y_{1}^{*2}}+\frac{16\sigma^{*}T_{\infty }^{3}}{3\kappa^{*}{\left(\rho c_{p}\right)}_{nf}}\frac{\partial^{2}T_{1}^{*}}{\partial y_{1}^{*2}}+\tau (D_{B}\frac{\partial C_{1}^{*}}{\partial y_{1}^{*}}\frac{\partial T_{1}^{*}}{\partial y_{1}^{*}}\\ +\frac{D_{T}}{T_{\infty }^{*}}{\left(\frac{\partial T_{1}^{*}}{\partial y_{1}^{*}}\right)}^{2})+\frac{\mu_{nf}}{{\left(\rho Cp\right)}_{nf}\left(1+\lambda_{1}\right)}\left[{\left(\frac{\partial u_{1}^{*}}{\partial y_{1}^{*}}\right)}^{2}+\lambda_{2}\left(u_{1}^{*}\frac{\partial u_{1}^{*}}{\partial y_{1}^{*}}\frac{\partial^{2}u_{1}^{*}}{\partial x_{1}^{*}\partial y_{1}^{*}}+v_{1}^{*}\frac{\partial u_{1}^{*}}{\partial y_{1}^{*}}\frac{\partial^{2}u_{1}^{*}}{\partial y_{1}^{*2}}\right)\right]\\ +\frac{\sigma_{nf}}{{\left(\rho Cp\right)}_{nf}}{\left(u_{1}^{*}B\left(x_{1}^{*}\right)-E\left(x_{1}^{*}\right)\right)}^{2}+\frac{Q_{e}^{*}\left(T_{s}^{*}-T_{\infty }^{*}\right)}{{\left(\rho Cp\right)}_{nf}}\exp\left(-n_{1}y_{1}^{*}\sqrt{\frac{(n+1)a}{2\nu }}{\left(x_{1}^{*}+b\right)}^{\frac{n-1}{2}}\right), \end{array}$$

(6) $$u_{1}^{*}\frac{\partial C_{1}^{*}}{\partial x_{1}^{*}}+v_{1}^{*}\frac{\partial C_{1}^{*}}{\partial y_{1}^{*}}=\frac{D_{T}}{T_{\infty }^{*}}\frac{\partial^{2}T_{1}^{*}}{\partial y_{1}^{*2}}+D_{B}\frac{\partial^{2}C_{1}^{*}}{\partial y_{1}^{*2}}-K_{r}\left(C_{1}^{*}-C_{\infty }^{*}\right),$$

(7) $$u_{1}^{*}\frac{\partial N_{1}^{*}}{\partial x_{1}^{*}}+v_{1}^{*}\frac{\partial N_{1}^{*}}{\partial y_{1}^{*}}+\left[\frac{\partial }{\partial y}\left(N\frac{\partial C_{1}^{*}}{\partial y_{1}^{*}}\right)\right]\frac{bW_{c}}{C_{w}-C_{\infty }}=D_{m}\frac{\partial^{2}N_{1}^{*}}{\partial y_{1}^{*2}}.$$

where $B\left(x_{1}^{*}\right)=B_{0}{\left(x_{1}^{*}+b\right)}^{\frac{n-1}{2}}$ is the applied magnetic field, $E\left(x_{1}^{*}\right)=E_{0}{\left(x_{1}^{*}+b\right)}^{\frac{n-1}{2}}$ is the applied electric field in a direction perpendicular to the elongating surface, $U_{s}\left(x_{1}^{*}\right)=a{\left(x_{1}^{*}+b\right)}^{n}$ is the velocity of the elongating surface at $y_{1}^{*}=A{\left(x_{1}^{*}+b\right)}^{\frac{\left(1-n\right)}{2}}$, and *n* is the power index.

### 2.3. Similarity Transformations

In fluid mechanics, the majority of known exact solutions are similarity solutions in which the number of independent variables is reduced by one or more. Similarity solutions are typically asymptotic problem solutions used to gain physical insight into the characteristics of complex fluid movements. These solutions represent the physical, as well as dynamic, and thermal parameters of the actual situation and their influence. Similarity transformations introduced to obtain the dimensionless form of governing equation for this Jeffrey fluid model are

(8) $$\begin{array}{cc} u_{1}^{*} & =\frac{\partial \psi }{\partial y_{1}^{*}}=U_{s}\left(x_{1}^{*}\right)G^{\prime }\left(\zeta \right),v_{1}^{*}=-\frac{\partial \psi }{\partial x_{1}^{*}}=-\sqrt{\frac{(n+1)\nu a}{2}}{\left(x_{1}^{*}+b\right)}^{\frac{n-1}{2}}\left(G\left(\zeta \right)+\eta \frac{n-1}{n+1}G^{\prime }\left(\zeta \right)\right),\\ & \theta =\frac{T_{1}^{*}-T_{\infty }^{*}}{T_{s}^{*}-T_{\infty }^{*}},\phi =\frac{C_{1}^{*}-C_{\infty }^{*}}{C_{s}^{*}-C_{\infty }^{*}},\xi =\frac{N_{1}^{*}-N_{\infty }^{*}}{N_{s}^{*}-N_{\infty }^{*}},\zeta =y_{1}^{*}\sqrt{\frac{n+1}{2}\frac{a}{\nu }}{\left(x_{1}^{*}+b\right)}^{\frac{n-1}{2}},\\ & \psi =G\left(\zeta \right)\sqrt{\frac{2}{n+1}\nu a}{\left(x_{1}^{*}+b\right)}^{\frac{n+1}{2}}. \end{array}$$

After employing the similarity transformations, we obtain the following governing equations:

(9) $$\begin{array}{c} G^{\prime \prime \prime }\left(\zeta \right)+\left(1+\beta_{1}\right)\frac{\varepsilon_{2}}{\varepsilon_{1}}\left[G^{\prime \prime }\left(\zeta \right)G\left(\zeta \right)-\frac{2n}{n+1}G^{\prime 2}\left(\zeta \right)\right]+\left(1+\beta_{1}\right)\frac{\varepsilon_{5}}{\varepsilon_{1}}\left[M\left(E_{1}-G^{\prime }\left(\zeta \right)\right)\right]\\ +\left(1+\beta_{1}\right)\frac{\varepsilon_{2}\varepsilon_{4}}{\varepsilon_{1}}\left[Gr\theta \left(\zeta \right)+Gc\phi \left(\zeta \right)-Nc\xi \left(\zeta \right)\right]\\ +\beta_{2}\left[\frac{3n-1}{2}{\left(G^{\prime \prime }\left(\zeta \right)\right)}^{2}+\left(n-1\right)G^{\prime }\left(\zeta \right)G^{\prime \prime \prime }\left(\zeta \right)-\frac{n+1}{2}G\left(\zeta \right)G^{iv}\left(\zeta \right)\right]=0, \end{array}$$

(10) $$\begin{array}{c} \theta^{\prime \prime }\left(\zeta \right)+Pr\left[Nt\theta^{\prime 2}\left(\zeta \right)+Nb\theta^{\prime }\left(\zeta \right)\phi^{\prime }\left(\zeta \right)\right]+\frac{\varepsilon_{3}}{\left(\varepsilon_{6}+Nr\right)}Pr\left[G\left(\zeta \right)\theta^{\prime }\left(\zeta \right)\right]\\ +\frac{\varepsilon_{5}}{\left(\varepsilon_{6}+Nr\right)}\left[PrEcM{\left(G^{\prime }\left(\zeta \right)-E_{1}\right)}^{2}\right]+\frac{1}{\left(\varepsilon_{6}+Nr\right)}Pr\left[Q_{e}\exp\left(-n_{1}\zeta \right)\right]+\frac{PrEc\varepsilon_{1}}{\left(1+\beta_{1}\right)\left(\varepsilon_{6}+Nr\right)}\\ \times \left[G^{\prime \prime 2}\left(\zeta \right)+\beta_{2}\left(\frac{3n-1}{2}G^{\prime }\left(\zeta \right){\left(G^{\prime \prime }\left(\zeta \right)\right)}^{2}-\left(\frac{n+1}{2}\right)G\left(\zeta \right)G^{\prime \prime }\left(\zeta \right)G^{\prime \prime \prime }\left(\zeta \right)\right)\right]=0, \end{array}$$

(11) $$\phi^{\prime \prime }\left(\zeta \right)+LeG\left(\zeta \right)\phi^{\prime }\left(\zeta \right)+\frac{Nt}{Nb}\theta^{\prime \prime }\left(\zeta \right)-K\phi =0,$$

(12) $$\xi^{\prime \prime }\left(\zeta \right)+LbG\left(\zeta \right)-Pe\left[\phi^{\prime \prime }\left(\zeta \right)\left(\xi +\delta_{N}\right)+\xi^{\prime }\left(\zeta \right)\phi^{\prime }\left(\zeta \right)\right]=0.$$

where $\varepsilon_{1}=\frac{1}{{\left(1-\varphi \right)}^{2.5}}$, $\varepsilon_{2}=1-\varphi +\varphi \left(\frac{\rho_{p}}{\rho_{f}}\right)$, $\varepsilon_{3}=1-\varphi +\varphi \left(\frac{\rho C_{p}}{\rho C_{f}}\right)$, $\varepsilon_{4}=1-\varphi +\varphi \left(\frac{\rho \beta_{p}}{\rho \beta_{f}}\right)$, $\varepsilon_{5}=1+\frac{3(\sigma_{p}-\sigma_{f})\varphi }{\left(\sigma_{p}+2\sigma_{f}\right)-\left(\sigma_{p}-\sigma_{f}\right)\varphi }$, and $\varepsilon_{6}=\frac{\left(\kappa_{p}+2\kappa_{f}+2\varphi \left(\kappa_{p}-\kappa_{f}\right)\right)}{\left(\kappa_{p}+2\kappa_{f}-2\varphi \left(\kappa_{p}-\kappa_{f}\right)\right)}$. The dimensionless parameters obtained in the present fluid model are mentioned in the Table 1.

### 2.4. Boundary Conditions

The boundary conditions of the flow system are ([54])

$$u_{1}^{*}=U_{s}\left(x_{1}^{*}\right),\;v_{1}^{*}=0,\;T_{1}^{*}=T_{s}^{*},\;C_{1}^{*}=C_{s}^{*},\;N_{1}^{*}=N_{s}^{*},\;at\;y_{1}^{*}=A{\left(x_{1}^{*}+b\right)}^{\frac{1-n}{2}},$$

(13) $$u_{1}^{*}\to 0,\;T_{1}^{*}\to T_{\infty }^{*},\;C_{1}^{*}\to C_{\infty }^{*},\;N_{1}^{*}\to N_{\infty }^{*},\;as\;y_{1}^{*}\to \infty .$$

On employing the similarity transformation mentioned in Equation (8), the nondimensionalized form of the boundary conditions is

(14) $$G\left(\zeta \right)=\alpha \frac{1-n}{1+n},\;G^{\prime }\left(\zeta \right)=1,\;\theta \left(\zeta \right)=1,\;\phi \left(\zeta \right)=1,\;\xi \left(\zeta \right)=1\;\;\mathrm{at}\;\zeta =0,$$

(15) $$G^{\prime }\left(\zeta \right)\to 0,\;\theta \left(\zeta \right)\to 0,\;\phi \left(\zeta \right)\to 0,\;\xi \left(\zeta \right)\to 0\;\;\mathrm{as}\;\zeta \to \infty .$$

### 2.5. Numerical Methodology

**Figure 2:**
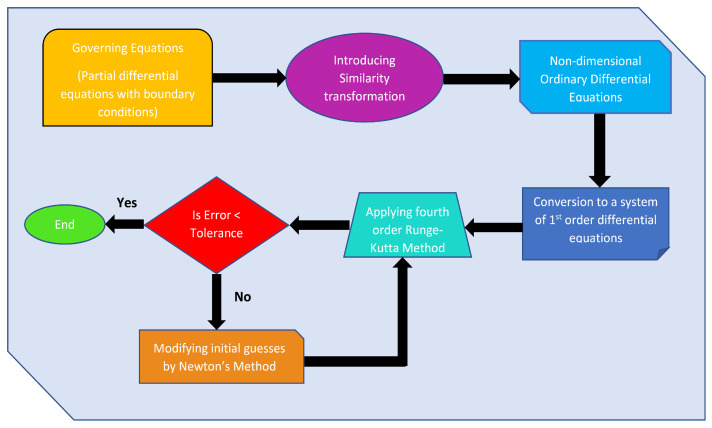
Flowchart presenting numerical methodology.

This section uses a numerical methodology to obtain the solution of the nondimensional higher-order coupled ODEs. In this methodology, higher-order ODEs are converted into a system of first-order ODEs with corresponding boundary conditions and then solved using the RK 4th-order method with shooting technique. To implement the RK 4th-order method, we need four initial conditions for the momentum equation, two initial conditions for the energy equation, two initial conditions for the concentration equation, and two for the equation of concentration of microorganisms. However, we only have two initial conditions given for the momentum equation and one initial condition given for the energy, concentration, and microorganism distribution equation. Consequently, the remainder of them are derived by updating the original predictions using Newton’s approach. The flow chart illustrating the numerical methodology used in the present analysis is shown in Figure 2. Let us assume

(16) $$\left\{\begin{array}{c} G=F,\;G^{\prime }=F_{1},\;G^{\prime \prime }=F_{2},\;G^{\prime \prime \prime }=F_{3},\\ \theta =F_{4},\;\theta^{\prime }=F_{5},\;\theta^{\prime \prime }=F_{6},\\ \phi =F_{7},\;\phi^{\prime }=F_{8},\;\phi^{\prime \prime }=F_{9},\\ \xi =F_{10},\;\xi^{\prime }=F_{11}, \end{array}\right.$$

The system of first-order differential equation is given by

(17) $$\left\{\begin{array}{c} F^{\prime }=F_{1},\\ F_{1}^{\prime }=F_{2},\\ F_{2}^{\prime }=F_{3},\\ F_{3}^{\prime }=\frac{2F_{3}}{\left(n+1\right)\beta_{2}F}+\frac{2\left(1+\beta_{1}\right)\varepsilon_{2}}{\beta_{2}\left(n+1\right)F\varepsilon_{1}}\left[FF_{2}-\frac{2n}{n+1}F_{1}^{2}\right]+\frac{2\varepsilon_{5}\left(1+\beta_{1}\right)}{\varepsilon_{1}\beta_{2}\left(n+1\right)F}M\left(E_{1}-F_{1}\right)+\frac{2\varepsilon_{2}\varepsilon_{4}\left(1+\beta_{1}\right)}{\varepsilon_{1}\beta_{2}\left(n+1\right)F}[GrF_{4}+GcF_{7}\\ \;\;\;\;\;\;\;\;\;\;\;\;\;\;\;\;\;\;\;\;\;\;\;\;\;\;\;\;\;\;\;\;\;\;\;\;\;\;\;\;\;\;\;\;\;\;\;\;\;\;\;\;\;\;\;\;\;\;\;\;\;\;\;\;\;-NcF_{10}]+\frac{2}{(n+1)F}\left[\left(\frac{3n-1}{2}\right){\left(F_{2}\right)}^{2}+\left(n-1\right)F_{1}F_{3}\right]\\ F_{4}^{\prime }=F_{5},\\ F_{5}^{\prime }=F_{6}=-\frac{\varepsilon_{3}}{\varepsilon_{6}}\frac{Pr}{\left(1+Nr\right)}\left[FF_{5}\right]-Pr\left[NbF_{5}F_{8}+NtF_{5}^{2}\right]+\frac{\varepsilon_{5}}{\varepsilon_{6}}PrMEc{\left(F_{1}-E_{1}\right)}^{2}+\frac{1}{\varepsilon_{6}}Pr\left[Q_{e}exp\left(-n_{1}\eta \right)\right]\\ \;\;\;\;\;\;\;\;\;\;\;\;\;\;\;\;\;\;\;\;\;\;\;\;\;\;\;\;\;\;\;\;\;\;\;\;\;\;\;\;\;\;\;\;\;\;\;\;\;\;\;\;\;\;\;\;\;\;\;\;\;\;\;\;\;\;\;\;\;+\frac{PrEc\varepsilon_{1}}{\left(1+\beta_{1}\right)\epsilon_{6}}\left[F_{2}^{2}+\beta_{2}\left(\frac{3n-1}{2}FF_{2}^{2}-\frac{n+1}{2}FF_{2}F_{3}\right)\right]\\ F_{7}^{\prime }=F_{8},\\ F_{8}^{\prime }=F_{9}=-\frac{Nt}{Nb}F_{6}-LeFF_{8}+LeKF_{7},\\ F_{10}^{\prime }=F_{11},\;\\ F_{11}^{\prime }=-LbFF_{11}+Pe\left[F_{9}\left(F_{10}+\delta_{N}\right)+F_{8}F_{11}\right] \end{array}\right.$$

and the corresponding boundary conditions become

(18) $$\left\{\begin{array}{c} F\left(\zeta \right)=\alpha \frac{1-n}{1+n},\;F_{1}\left(\zeta \right)=1,\;F_{4}\left(\zeta \right)=1,\;F_{7}\left(\zeta \right)=1,\;F_{10}\left(\zeta \right)=1\;\;\mathrm{at}\;\zeta =0,\;\\ F_{1}\left(\zeta \right)\to 0,\;F_{4}\left(\zeta \right)\to 0,\;F_{7}\left(\zeta \right)\to 0,\;F_{10}\left(\zeta \right)\to 0\;\;\mathrm{as}\;\zeta \to \infty . \end{array}\right.$$

## 3. Result and Discussion

The numerical findings computed with the help of the above methodology are discussed in this section for various emergent parameter values. The remarkable impacts of pertinent parameters are shown in graphical forms in terms of fluid velocity $G^{\prime }\left(\zeta \right)$, dimensionless temperature θ(ζ), dimensionless concentration of the fluid ϕ(ζ), microorganism density ξ(ζ), entropy generation number $N_{s}$, Bejan number Be, drag coefficient $C_{f}$, local Nusselt number $Nu_{x}$, Sherwood number $Sh_{x}$, and the local gyrotactic microorganism density $Nn_{x}$. The thermophysical parameters of nanofluid are mentioned using Table 2. Table 3 depicts the thermophysical properties of Copper nanoparticles, polyvinyl alcohol (PVA), and water. The default values of the parameters considered in the present analysis are listed in Table 4.

**Figure 3:**
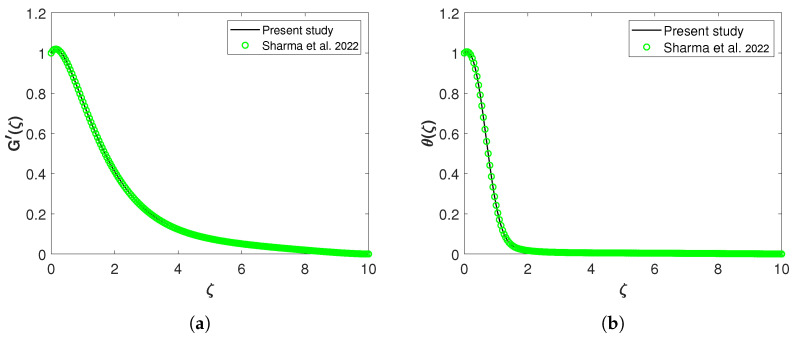
Comparative analysis of (**a**) velocity profile $G^{\prime }\left(\zeta \right)$ for Gc=1 and (**b**) temperature profile θ(ζ) for Pr=7. Sharma et al. [27].

The verification of the numerical scheme employed is mandatory, and therefore, the study by Sharma et al. [29] is used to validate the results obtained from the above methodology. In the absence of a few physical factors, the current model reduces to that of [29]. The influence of microorganisms, solar radiations, and nanoparticle volume fraction in the present analysis and the effect of thermal heat source and activation energy in [29] was disregarded to verify the results with those of Sharma et al. [29]. Figure 3a,b displays validation plots, with Figure 3a displaying the validation graph of velocity profiles and Figure 3b showing the validation graph of temperature profiles. Based on these Figures, the current analysis’s findings are in excellent accord with the work of Sharma et al. [29].

**Figure 4:**
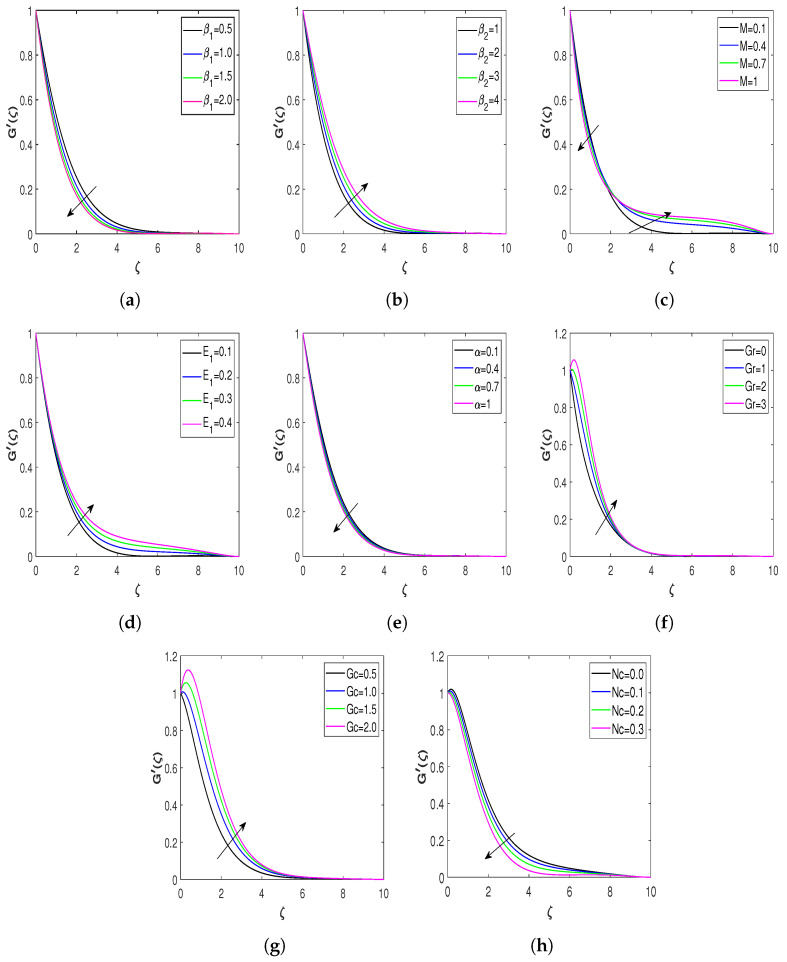
Nondimensional velocity profiles for different values of influential parameters. (**a**) Velocity profile against $\beta_{1}$; (**b**) velocity profile against $\beta_{2}$; (**c**) velocity profile against *M*; (**d**) velocity profile against $E_{1}$; (**e**) velocity profile against α; (**f**) velocity profile against Gr; (**g**) velocity profile against Gc; and (**h**) velocity profile against Nc.

The plots of fluid velocity against the different physical parameters are depicted in Figure 4a–h. The physical impact of $\beta_{1}$ on fluid velocity is highlighted in Figure 4a. This Figure illustrates that fluid velocity diminishes with an increment in $\beta_{1}$. Higher values of retardation time enhance the momentum boundary layer of the flow, since retardation time varies inversely with the Deborah number $\beta_{1}$. So, the escalating values of $\beta_{1}$ reduce the fluid velocity. Figure 4b represents the fluid’s velocity behavior corresponding to Deborah number $\beta_{2}$. The velocity of the fluid rises with an increment in $\beta_{2}$. The boundary layer of the flow field improves as the retardation time parameter is increased. Therefore, the velocity profile of the flow field enhances with the augmenting values of $\beta_{2}$. Figure 4c demonstrates the magnetic field’s effect on fluid velocity. Intriguingly, this Figure indicates that the velocity of the fluid initially drops as the *M* increases and then exhibits the opposite behavior as the similarity variable reaches a specific value. This outcome is consistent with the findings of [7]. The increased magnetic field’s Lorentz force serves as a frictional force. This frictional force resists the fluid flow, but when the magnetic field strengthens in the presence of an electric field, the electric field produces an accelerating force. Thus, the velocity profile changes its behavior close to the stretching surface and increases after a certain distance from the wall. Figure 4d represents the influence of $E_{1}$ on the fluid velocity, demonstrating that fluid velocity increases as $E_{1}$ increases. Increasing values of $E_{1}$ accelerate the nanofluid flow, enhancing the fluid’s velocity. The fluid’s velocity profiles for the thickness parameter α are displayed in Figure 4e. The increasing wall thickness parameter results in a thicker momentum boundary layer, retarding the fluid’s velocity. Figure 4f represents the fluid velocity variation corresponding to the Grashof number Gr, which reveals that the velocity of the fluid boosts with Gr. Physically, the growing values of Gr increase the temperature gradient and improve the buoyancy force, which accelerates the fluid flow. Figure 4g depicts the effect of Gc and the flow field velocity. This figure reveals that augmenting values of Gc enhance the fluid velocity because of a stronger buoyancy force. This force produces a pressure gradient in the flow field, which causes fluid acceleration. Hence, fluid velocity raises with escalating values of Gc. The effect of the velocity profile against bioconvection Rayleigh number Nc is portrayed in Figure 4h. This figure shows that higher bioconvection Rayleigh number Nc values resist the upward nanofluid flow.

**Figure 5:**
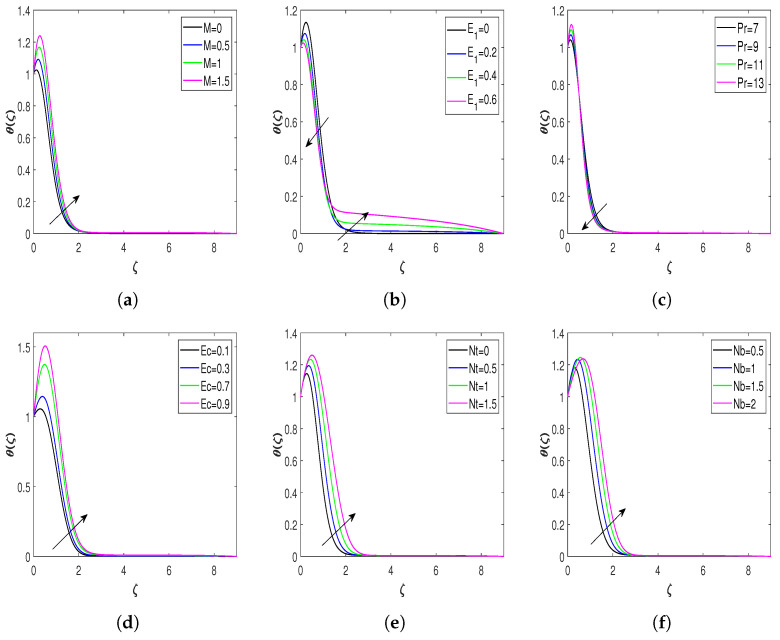

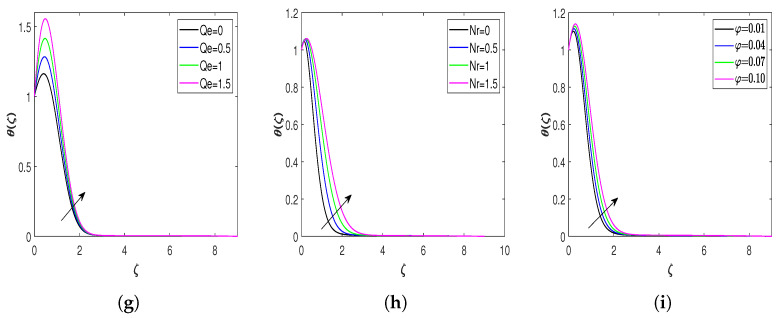
Nondimensional temperature profiles for different values of influential parameters. (**a**) Temperature profile against *M*; (**b**) temperature profile against $E_{1}$; (**c**) temperature profile against Pr; (**d**) temperature profile against Ec; (**e**) temperature profile against Nt; (**f**) temperature profile against Nb; (**g**) temperature profile against $Q_{e}$; (**h**) temperature profile against Nr; and (**i**) temperature profile against φ.

The remarkable impact of numerous physical parameters on the nondimensional temperature of the fluid is presented in Figure 5a–h. Fluid temperature profiles for parameter *M* are shown in Figure 5a. A rise in the values of *M* improves the fluid’s temperature, since an increase in the magnetic field produces a stronger Lorentz force. Figure 5b portrays the influence of $E_{1}$ on the nondimensional temperature of the fluid. The temperature profiles show declination up to a particular value of the similarity variable ζ but then modify its behavior and depict an inclining effect as it approaches farther from the wall of the stretching surface. The heat transfer features via Prandtl number Pr are illustrated in Figure 5c. The temperature of the flow field diminishes with the augmenting values of Pr. The flow becomes viscous-dominant for higher Prandtl number values, and the thermal boundary layer becomes thinner due to viscous dominance. Figure 5d represents the variation of fluid temperature versus Ec. The characterization of viscous heat dissipation is defined by Ec. A higher Eckert number causes more friction on the adjacent fluid layers. An increase in frictional forces magnifies the flow field’s internal heat energy. Therefore, the temperature rises with Ec. Figure 5e highlights the behavior of Nt on the fluid temperature. The growth in the values of Nt modifies the fluid temperature. Thermophoresis is the force responded to by suspended particles of the fluid owing to its thermal gradient. An increase in thermal gradient enhances the flow field’s temperature. Therefore, fluid temperature enhances with growing values of Nt. Figure 5f displays the impact of Nb on the fluid temperature. This graph illustrates that a rise in Nb raises the temperature. The random motion of particles under suspension in liquid is called Brownian motion. A particle changes its path when it collides with another particle. Furthermore, these collisions cause a random or zigzag motion, and the collision involves the transfer of energy between the particles. Therefore, the augmenting values of Nb improve the temperature profile. Variation of the exponential heat source Qe with the temperature of the flow field is shown in Figure 5g. The escalating values of Qe raise the fluid temperature. Generally, an exponential heat source energizes the flow field, improving the fluid temperature. Figure 5h reveals the impact of Nr on the temperature, which clarifies that an increasing radiation parameter increases the temperature of the fluid. The thermal radiation provides thermal energy to the stretching surface, and then the temperature profile of the fluid enhances due to the conduction between the surface and fluid. Figure 5i represents the influence of φ on the fluid temperature. As per this figure, an increase in the volume fraction improves heat transfer. An increment in φ improves the Jeffrey nanofluid’s thermal conductivity, which improves the flow’s thermal boundary layer. Hence, thermal profiles escalate with the volume fraction of the nanoparticle.

**Figure 6:**
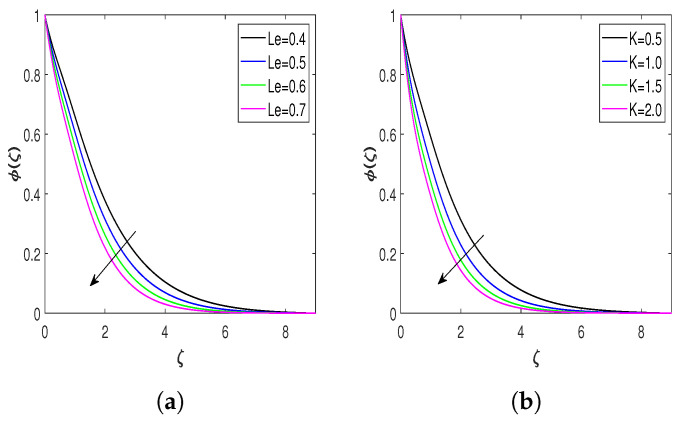
Nondimensional concentration profiles for flow parameters. (**a**) Concentration profile against Le and (**b**) concentration profile against *K*.

The influence of Lewis number Le and chemical reaction parameter *K* on the concentration of the fluid is shown in Figure 6a,b. The behavior of Le concerning the fluid’s concentration is displayed in Figure 6a, which declares that the concentration of the flow field diminishes with the growing values of Le. Higher values of Le reduce the solute diffusivity, which drops the concentration of the nanofluid, and the mass transfer rate accelerates at the stretching surface. Therefore, the concentration of the nanofluid reduces with Le. The effect of *K* on the fluid’s concentration is highlighted in Figure 6b. Increasing values of *K* decelerate mass diffusivity and diminish the concentration boundary layer in the flow field. Hence, the fluid’s concentration decreases with *K*, which is in good agreement with [29].

**Figure 7:**
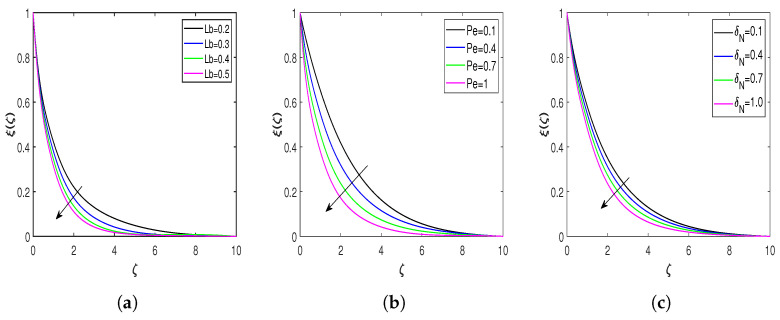
Distribution of microorganisms for different flow parameters. (**a**) Microorganism distribution for Lb; (**b**) Microorganism distribution for Pe; (**c**) Microorganism distribution for $\delta_{N}$.

The behavior of the density of microorganism distribution ξ against various physical parameters are displayed in Figure 7a–c. The Lb features on the density of microorganisms are depicted in Figure 7a. The microorganisms’ concentration distribution reduces with Lb. Due to the fact that a greater Lb indicates a weaker Brownian motion diffusion coefficient, swimming microorganisms have a relatively shallow penetration depth. Hence, the concentration of microorganism distribution diminishes with Lb, and this result is in validation with the result in the previously published literature [55]. The variation of concentration of motile microorganism distribution versus Peclet number Pe is plotted in Figure 7b. There is a decline in the gyrotactic microorganisms’ concentration with a rise in the values of Pe. The impact of $\delta_{N}$ on the motile microorganisms’ density is described in Figure 7c. The motile microorganism density decreases with the enhancement in the microorganism difference parameter. This behavior of $\delta_{N}$ is precisely similar to the result in the literature [49].

### 3.1. Entropy Generation Model

Entropy generation of this Jeffrey fluid model due to momentum, heat transfer, mass transfer, and density of microorganism distribution is given by

(19) $$\begin{array}{cc} E_{g} & =\left(\kappa_{nf}+\frac{16\sigma^{*}T_{\infty }^{3}}{3\kappa^{*}{\left(\rho c_{p}\right)}_{nf}}\right)\frac{{\left(\nabla T_{f}\right)}^{2}}{{\left(T_{\infty }^{*}\right)}^{2}}+\mu_{nf}\frac{F}{T_{\infty }^{*}}+\frac{\sigma_{nf}}{{\left(T_{\infty }^{*}\right)}^{2}}{\left(u_{1}^{*}B-E\right)}^{2}+RD\frac{{\left(\nabla C_{f}\right)}^{2}}{C_{\infty }^{*}}\\ & +RD\frac{\nabla C_{f}\nabla T_{f}}{T_{\infty }^{*}}+RD\frac{{\left(\nabla N_{f}\right)}^{2}}{N_{\infty }^{*}}+RD\frac{\nabla N_{f}\nabla T_{f}}{T_{\infty }^{*}}. \end{array}$$

The mathematical expression of entropy generation of this fluid model is

(20) $$\begin{array}{c} E_{g}=\frac{1}{{\left(T_{\infty }^{*}\right)}^{2}}\left(\kappa_{nf}+\frac{16\sigma^{*}T_{\infty }^{3}}{3\kappa^{*}{\left(\rho c_{p}\right)}_{nf}}\right){\left(\frac{\partial T_{1}^{*}}{\partial y_{1}^{*}}\right)}^{2}\\ +\frac{\mu_{nf}}{T_{\infty }^{*}\left(1+\lambda_{2}\right)}\left[{\left(\frac{\partial u_{1}^{*}}{\partial y_{1}^{*}}\right)}^{2}+\lambda_{1}\left(u_{1}^{*}\frac{\partial u_{1}^{*}}{\partial y_{1}^{*}}\frac{\partial^{2}u_{1}^{*}}{\partial x_{1}^{*}\partial y_{1}^{*}}+v_{1}^{*}\frac{\partial u_{1}^{*}}{\partial y_{1}^{*}}\frac{\partial^{2}u_{1}^{*}}{\partial y_{1}^{*2}}\right)\right]+\frac{\sigma_{nf}}{{\left(T_{\infty }^{*}\right)}^{2}}{\left(u_{1}^{*}B-E\right)}^{2}\\ +\frac{RD_{b}}{C_{\infty }^{*}}{\left(\frac{\partial C_{1}^{*}}{\partial y_{1}^{*}}\right)}^{2}+\frac{RD_{b}}{T_{\infty }^{*}}\left(\frac{\partial C_{1}^{*}}{\partial y_{1}^{*}}\right)\left(\frac{\partial T_{1}^{*}}{\partial y_{1}^{*}}\right)+\frac{RD_{b}}{N_{\infty }^{*}}{\left(\frac{\partial N_{1}^{*}}{\partial y_{1}^{*}}\right)}^{2}+\frac{RD_{b}}{T_{\infty }^{*}}\left(\frac{\partial N_{1}^{*}}{\partial y_{1}^{*}}\right)\left(\frac{\partial T_{1}^{*}}{\partial y_{1}^{*}}\right). \end{array}$$

Mathematical expression of the entropy generation into nondimensionalized form:

(21) $$\begin{array}{c} N_{s}=\left(\varepsilon_{6}+Nr\right)\delta {\theta^{\prime }}^{2}+\varepsilon_{1}\frac{PrEc}{\left(1+\beta_{1}\right)}\left[{G^{\prime \prime }}^{2}+\beta_{2}\left(\frac{3n-1}{2}G^{\prime }{G^{\prime \prime }}^{2}-\frac{n+1}{2}GG^{\prime \prime }G^{\prime \prime \prime }\right)\right]+\\ \varepsilon_{5}MPrEc{\left(G^{\prime }-E_{1}\right)}^{2}+L\theta^{\prime }\phi^{\prime }+L^{*}\frac{1}{\delta \delta_{N}}{\xi^{\prime }}^{2}+L\frac{\delta_{1}}{\delta }{\phi^{\prime }}^{2}+L^{*}\theta^{\prime }\xi^{\prime }.\;\;\;\;\;\;\; \end{array}$$

The mathematical expression of the Bejan number is

(22) $$Be=\frac{\left(\varepsilon_{6}+Nr\right)\delta {\theta^{\prime }}^{2}+L\theta^{\prime }\phi^{\prime }+L^{*}\frac{1}{\delta \delta_{N}}{\xi^{\prime }}^{2}+L\frac{\delta_{1}}{\delta }{\phi^{\prime }}^{2}+L^{*}\theta^{\prime }\xi^{\prime }}{N_{s}}.$$

**Figure 8:**
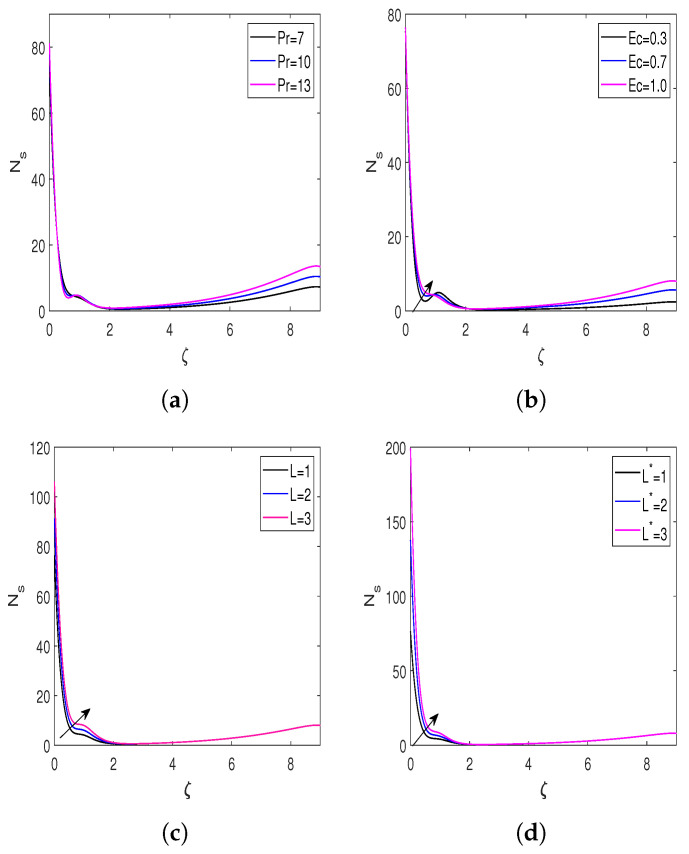
Variation in entropy for different flow parameters. (**a**) Entropy versus Pr; (**b**) entropy versus Ec; (**c**) entropy versus *L*; and (**d**) entropy versus $L^{*}$.

**Figure 9:**
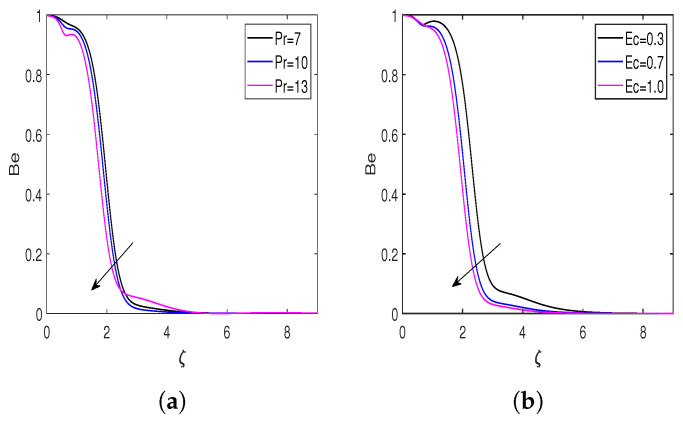

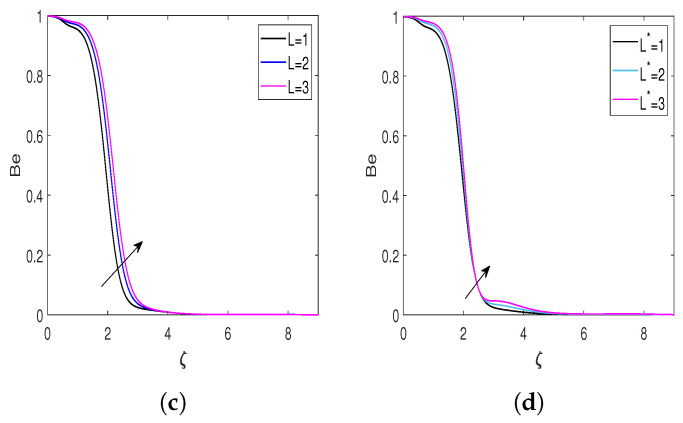
Bejan number profiles for different influential parameters. (**a**) Bejan number versus Pr; (**b**) Bejan number versus Ec; (**c**) Bejan number versus *L*; and (**d**) Bejan number versus $L^{*}$.

The impacts of the Prandtl number, Eckert number, diffusion parameter, and microorganism diffusion parameter on the entropy generation number ($N_{s}$) and Bejan number (Be) are presented in Figure 8 and Figure 9. The behavior of entropy $N_{s}$ for the Pr is displayed in Figure 8a, demonstrating that enhancing the Prandtl number escalates the entropy formation in the flow field. Figure 8b represents the impact of the Ec on the entropy generation, which declares that enhancement in the Ec improves the rate of entropy generation. A rise in Eckert’s number amplifies the difference between the enthalpy and kinetic energy of the flow boundary layer, hence increasing the flow field’s irreversibility. Hence, the rate of entropy generation enhances for the larger Eckert number. The behavior of entropy corresponding to the diffusion parameter is highlighted in Figure 8c. From this Figure, it is clear that the entropy rate enhances due to a rise in the diffusion parameter. The flow field becomes more disorderly as the diffusion parameter rises, which causes irreversibility to rise and, as a result, accelerates entropy formation. Figure 8d illustrates the entropy variation for the bioconvection diffusion parameter. Diffusion of bioconvection increases the system’s irreversibility. Hence, the higher values of $L^{*}$ increase the entropy generation rate. Figure 9a,b represents the Bejan number profiles for Eckert and Prandtl numbers. There is a decline in Be profiles with an increment in Pr and Ec. Figure 9c,d reveals the influence of the diffusion and bioconvection diffusion parameters on the Bejan number. According to these figures, increasing the values of *L* and $L^{*}$ enhances the Bejan number.

**Figure 10:**
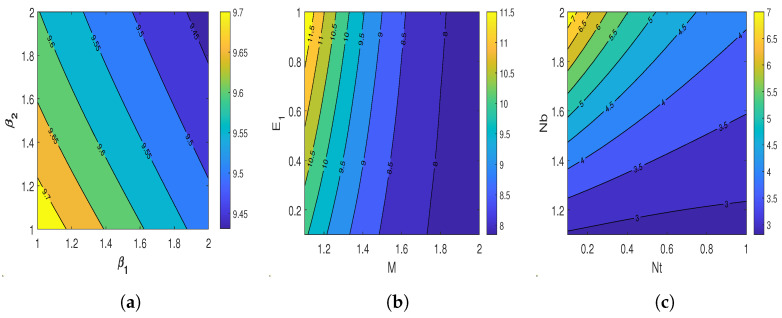

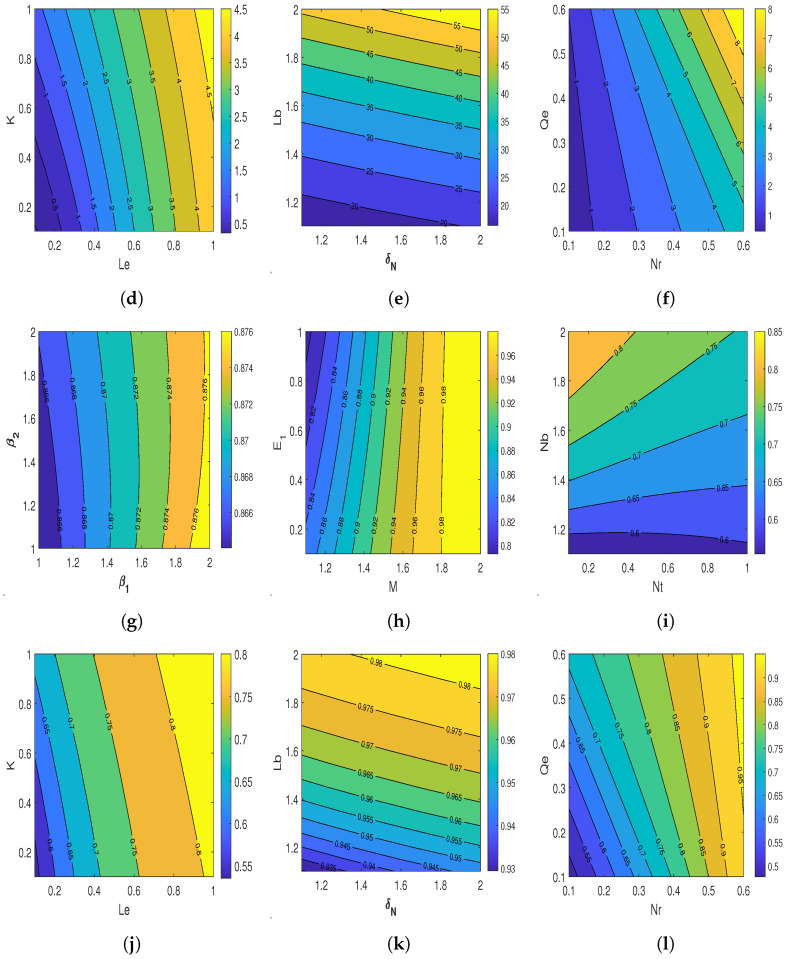
Contour plots illustrating the effect of various influential parameters on entropy generation and Bejan number. (**a**) Entropy via $\beta_{1}$ and $\beta_{2}$; (**b**) entropy via *M* and $E_{1}$; (**c**) entropy via Nt and Nb; (**d**) entropy via *K* and Le; (**e**) entropy via $\delta_{N}$ and Lb; (**f**) entropy via Nr and Qe; (**g**) Bejan number via $\beta_{1}$ and $\beta_{2}$; (**h**) Bejan number via *M* and $E_{1}$; (**i**) Bejan number via Nt and Nb; (**j**) Bejan number via *K* and Le; (**k**) Bejan number via $\delta_{N}$ and Lb; and (**l**) Bejan number via Nr and Qe.

Contour plots illustrate the impact of different influential flow parameters on entropy generation parameter $N_{s}$ and Bejan number Be in the Figure 10a–l. Figure 10a shows the variation of $\beta_{1}$ and $\beta_{2}$ on the entropy $N_{s}$. An increment in $\beta_{1}$ and $\beta_{2}$ reduces the entropy values. The behavior of $\beta_{1}$ and $\beta_{2}$ versus Be is displayed in Figure 10f. The Be rises with $\beta_{1}$ and $\beta_{2}$, but the growth rate of the Bejan number due to $\beta_{1}$ is more compared with $\beta_{2}$. The contour plot illustrating the effect of *M* and $E_{1}$ on entropy is shown in Figure 10b. An opposite effect on entropy is observed with *M* and $E_{1}$, i.e., an increment in *M* declines the entropy, while the increasing values of $E_{1}$ enhance the entropy. Figure 10g represents the behavior of *M* and $E_{1}$ on Be. The Bejan number diminishes for the higher values of $E_{1}$ and improves with the growth in *M*. The influence on entropy via Nt and Nb are depicted in Figure 10c. This figure declares that entropy increases with the escalating values of Nb, while it shows declination with Nt. The contour for the Bejan number showing the influence of Nt and Nb is highlighted in Figure 10h. According to this figure, larger values of Nt cause the Bejan number to decrease, but higher values of Nb cause the Bejan number to increase. The behavior of *K* and Le versus entropy is shown in Figure 10d. This figure declares that entropy declines with an increase in *K* and grows with an increase in Le. Figure 10i displays the variation in Bejan number with *K* and Le. Be values get reduced with increasing *K* and decreasing Le. Figure 10e shows the influence of $\delta_{N}$ and Lb on entropy formation. The entropy formation rate boosts due to an increase in both $\delta_{N}$ and Lb. Bejan number variations for $\delta_{N}$ and Lb are displayed in Figure 10j. The Bejan number also shows enhancement for augmenting values of $\delta_{N}$ and Lb. Figure 10f,l displays the impact of radiation parameter Nr and exponential heat source Qe on $N_{s}$ and Be, which shows that an increase in Nr and Qe increases both the entropy and Bejan number of the flow field.

### 3.2. Physical Quantities of Engineering Interest

Drag coefficient ($C_{f}$),

(23) $$C_{f}=\frac{\tau_{s}^{*}}{\rho {\left(U_{s}\right)}^{2}},\;\tau_{s}^{*}={\left.\frac{\mu_{nf}}{1+\lambda_{1}}\left[\frac{\partial u_{1}^{*}}{\partial y_{1}^{*}}+\lambda_{2}\left(u_{1}^{*}\frac{\partial^{2}u_{1}^{*}}{\partial x_{1}^{*}\partial y_{1}^{*}}+v_{1}^{*}\frac{\partial^{2}u_{1}^{*}}{\partial y_{1}^{*2}}\right)\right]\right|}_{y_{1}^{*}=A{\left(x_{1}^{*}+b\right)}^{\frac{1-n}{2}}},$$

Local Nusselt number microorganisms ($Nu_{x}$),

(24) $$Nu_{x}=\frac{\left(x_{1}^{*}+b\right)q_{s}}{\kappa_{nf}\left(T_{s}^{*}-T_{\infty }^{*}\right)},\;q_{s}=-{\left.\kappa_{nf}\frac{\partial T_{1}^{*}}{\partial y_{1}^{*}}\right|}_{y_{1}^{*}=A{\left(x_{1}^{*}+b\right)}^{\frac{1-n}{2}}},$$

Sherwood number ($Sh_{x}$)

(25) $$Sh_{x}=\frac{\left(x_{1}^{*}+b\right)q_{m}}{D_{B}\left(C_{s}^{*}-C_{\infty }^{*}\right)},\;q_{m}=-{\left.D_{B}\frac{\partial C_{1}^{*}}{\partial y}\right|}_{y_{1}^{*}=A{\left(x_{1}^{*}+b\right)}^{\frac{1-n}{2}}},$$

Local density of microorganisms ($Nn_{x}$),

(26) $$Nn_{x}=\frac{\left(x_{1}^{*}+b\right)q_{m}}{D_{B}\left(C_{s}^{*}-C_{\infty }^{*}\right)},\;q_{n}=-{\left.D_{n}\frac{\partial N_{1}^{*}}{\partial y}\right|}_{y_{1}^{*}=A{\left(x_{1}^{*}+b\right)}^{\frac{1-n}{2}}}.$$

Using similarity transformation variables, the nondimensionlized forms of the above quantities are given by

(27) $$\begin{array}{c} Re_{x}{}^{1/2}C_{f}={\left.\sqrt{\frac{n+1}{2}}\frac{1}{1+\beta_{1}}\left[G^{\prime \prime }\left(\eta \right)+\beta_{2}\left(\frac{3n-1}{2}G^{\prime }\left(\eta \right)G^{\prime \prime }\left(\eta \right)-\frac{n+1}{2}G\left(\eta \right)G^{\prime \prime \prime }\left(\eta \right)\right)\right]\right|}_{\eta =0},\\ Re_{x}{}^{-1/2}Nu_{x}=-{\left.\sqrt{\frac{n+1}{2}}\theta^{\prime }\left(\eta \right)\right|}_{\eta =0},\;Re_{x}{}^{-1/2}Sh_{x}=-{\left.\sqrt{\frac{n+1}{2}}\phi^{\prime }\left(\eta \right)\right|}_{\eta =0},\\ Re_{x}{}^{-1/2}Nn_{x}=-{\left.\sqrt{\frac{n+1}{2}}\xi^{\prime }\left(\eta \right)\right|}_{\eta =0}. \end{array}$$

**Figure 11:**
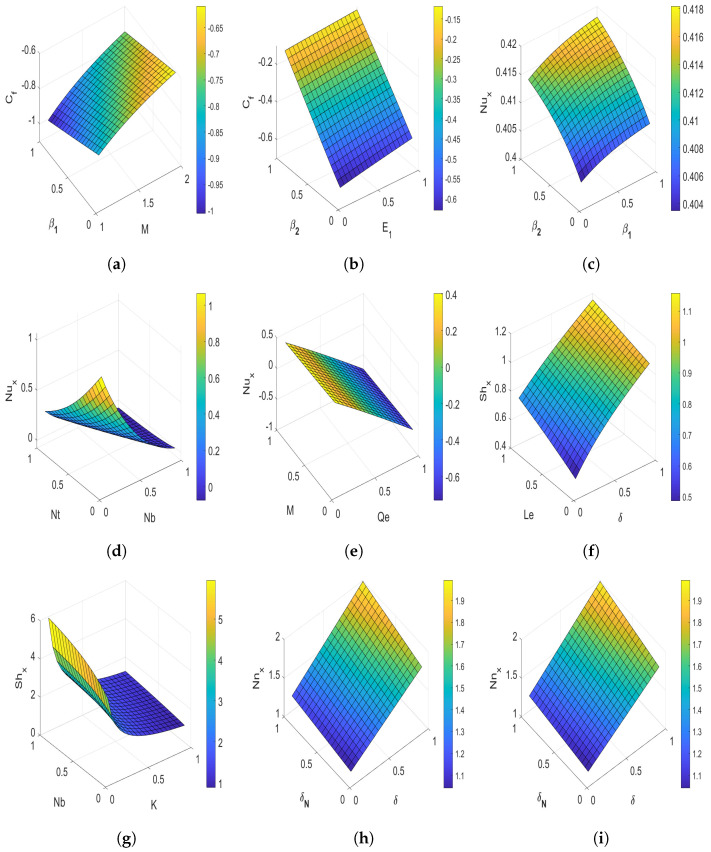
Surface plots displaying the effects of various influence parameters on $C_{f}$, $Nu_{x}$, $Sh_{x}$, and $Nn_{x}$. (**a**) $C_{f}$ versus *M* and $\beta_{1}$; (**b**) $C_{f}$ versus $E_{1}$ and $\beta_{2}$; (**c**) $Nu_{x}$ versus $\beta_{1}$ and $\beta_{2}$; (**d**) $Nu_{x}$ versus Nt and Nb; (**e**) $Nu_{x}$ versus *M* and Qe; (**f**) $Sh_{x}$ versus δ and Le; (**g**) $Sh_{x}$ versus *K* and Nb; (**h**) $Nn_{x}$ versus δ and $\delta_{N}$; and (**i**) $Nn_{x}$ versus Pe and Lb.

The drag coefficient ($C_{f}$), dimensionless parameter of heat transfer coefficient in terms of Nusselt number ($Nu_{x}$), dimensionless parameter of mass transfer coefficient in terms of Sherwood number ($Sh_{x}$), and the local density of the microorganisms ($Nn_{x}$) results are illustrated using surface plots. The effects of the magnetic field parameter *M* and Deborah number $\beta_{1}$ versus drag coefficient are plotted in Figure 11a. It is observed that the larger magnetic field parameter increases the drag coefficient, while the larger $\beta_{1}$ diminishes the drag coefficient. Figure 11b illustrates the surface plot of the electric field parameter $E_{1}$ and Deborah number $\beta_{2}$ versus drag coefficient. This plot declares that the drag coefficient improves with the escalation in $E_{1}$, but the effect of the $E_{1}$ is much less than that of Deborah number $\beta_{2}$. Figure 11c represents the Nusselt number results of Deborah numbers $\beta_{1}$ and $\beta_{2}$. It is noticed that the Nusselt number at the stretching surface reduces with growing values of $\beta_{1}$, while the heat transfer rate escalates with Deborah number $\beta_{2}$. Figure 11d reveals the behavior of the $Nu_{x}$ corresponding to Nt and Nb. This Figure clarifies that the Nusselt number diminishes with enhancement in both Nt and Nb. The nature of the *M* and Qe on the Nusselt number is presented in Figure 11e, which declares that an increment in Qe enhances $Nu_{x}$. In contrast, the growth in *M* reduces the Nusselt number. Figure 11f declares the effect of the $Sh_{x}$ for Le and δ. In this Figure, the Sherwood number escalates with the augmenting values of δ and Le. The variation of the Sherwood number versus Nb and *K* is displayed in Figure 11g. This figure reveals that higher values of Nb enlarge the Sherwood number, while the Sherwood number reduces with enhancing *K*. Figure 11h represents the effects of δ and $\delta_{N}$ on $Nn_{x}$. It is examined that the local density of the microorganisms rises with δ and $\delta_{N}$. The temperature difference parameter is more dominant than the microorganism difference parameter. Figure 11i illustrates the effect of Lb and Pe on $Nn_{x}$. As per this figure, it is seen that augmenting the values of bioconvection Lb and Pe enlarges the local density of the motile gyrotactic microorganisms. Moreover, the Lb is more dominant than the Peclet number Pe.

## 4. Conclusions

This study discusses the EMHD Jeffrey nanofluid flow through a nonlinear vertically stretching surface of nonuniform thickness, considering the effects of solar radiation and the existence of gyrotactic microorganisms and copper nanoparticles with polyvinyl alcohol–water base fluid. The influence of the source terms such as Joule heating, exponential heat source, viscous dissipation, and chemical reaction is analyzed. The equations governing the fluid flow and boundary conditions are nondimensionalized using suitable similarity transformations. Higher-order nondimensional ODEs are reduced to a system of first-order ODEs and then solved using the RK 4th-order method and the shooting technique. The following are the conclusions of this study:

- Deborah number $\beta_{1}$ diminishes the velocity profile, while Deborah number $\beta_{2}$ enhances the velocity profile.
- Fluid velocity shows enhancement for rising values of $E_{1}$, Gr, and Gc, while fluid velocity decays for augmenting values of *M*, α, and Nc.
- The increment in *M*, $E_{1}$, Ec, Nt, Nb, Qe, and φ enhances the temperature profile, whereas the temperature profile decays for the magnifying values of Pr.
- The concentration profile decays for increasing values of Le and *K*.
- The rate of entropy increases with an increment in Pr and Ec, while the Bejan number shows declination.
- Both the entropy formation rate and Bejan number enhance with the increment diffusion parameter *L* and bioconvection diffusion parameter $L^{*}$.
- Drag coefficient improves with the growing values of *M*, $E_{1}$, and $\beta_{2}$, while the drag coefficient reduces with an increase in $\beta_{1}$.
- Nusselt number enlarges with the enhancement in $\beta_{2}$, Ec, and Qe. Moreover, it diminishes with a higher $\beta_{1}$, Pr, Nt, Nb, and *M*.
- Sherwood number escalates with the augmenting values of δ and Le. Moreover, it will reduce with augmenting values of *K*.
- $Nn_{x}$ improves with the growth in δ, Lb, and Pe, while it decreases with $\delta_{N}$.

The previous literature is limited to the entropy generation of the Jeffrey nanofluid flows in heat and mass transfer processes over the stretching surfaces of uniform thickness. No attempts have been made to analyze the entropy generation of the EMHD Jeffrey fluid flow with motile gyrotactic microorganisms via vertical surfaces stretching nonlinearly with nonuniform thickness under the influence of solar radiations. Therefore, this study investigates entropy generation and solar radiation effects in the presence of motile gyrotactic microorganisms and copper nanoparticles with polyvinyl alcohol–water as the base fluid, as well as the influence of the source terms, such as viscous dissipation, Ohmic heating, exponential heat source, and chemical reaction of the EMHD Jeffrey fluid flow past a vertical nonlinearly elongating surface of nonuniform thickness. This research could aid in constructing solar heat engines, solar thermochemical heat pumps, solar ponds, and household solar water heaters, among others. The mechanism of bioconvection will improve the stability of nanoparticles. The motile gyrotactic microorganisms are crucial, because a better liquid mixture is required for a better biological process. Entropy production improves system performance, updates systematic presentations, and destroys energy. So, this study can be helpful for such types of applications.

Optimizing the heat transfer in solar energy systems is a peculiar feature of storing thermal energy in the maximum possible amount from solar radiation. In this direction, nanofluids are proved to be very useful in improving the heat transfer properties of base fluids. In the future, the present work can be extended by using hybrid and ternary nanofluids, using other base fluids as heat transfer fluids, which may result in better heat transfer characteristics. A sensitivity analysis to determine the effective parameters affecting the heat transfer of hybrid nanofluid flow in the parabolic trough collectors in different base fluids can be performed.

## Figures and Tables

**Table 1:** Dimensionless parameter of the fluid model ([29,54]).

Magnetic field parameter, $M=\frac{2\sigma B_{0}^{2}}{\rho_{f}a\left(n+1\right)}$	Electric field parameter, $E_{1}=\frac{E_{0}}{B_{0}a{\left(x_{1}^{*}+b\right)}^{n}}$
Grashof number, $Gr=\frac{2g\beta_{T}\left(T_{s}^{*}-T_{\infty }^{*}\right)}{a^{2}\left(n+1\right){\left(x_{1}^{*}+b\right)}^{2n-1}}$	Solutal Grashof number, $Gc=\frac{2g\beta_{C}\left(C_{s}^{*}-C_{\infty }^{*}\right)}{a^{2}\left(n+1\right){\left(x_{1}^{*}+b\right)}^{2n-1}}$
Bioconvection Rayleigh parameter, $Nc=\frac{2g\beta_{N}\left(N_{s}^{*}-N_{\infty }^{*}\right)}{a^{2}\left(n+1\right){\left(x_{1}^{*}+b\right)}^{2n-1}}$	Radiation parameter, $Nr=\frac{16\sigma^{*}T_{\infty }^{*3}}{3\kappa_{f}\kappa^{*}}$
Eckert number, $Ec=\frac{U_{s}^{2}}{C_{p}\left(T_{s}^{*}-T_{\infty }^{*}\right)},$	Prandtl number, $Pr=\frac{\mu C_{p}}{\kappa },$
Temperature difference parameter, $\delta =\frac{T_{s}^{*}-T_{\infty }^{*}}{T_{\infty }^{*}}$	Exponential heat source parameter, $Q_{e}=\frac{2Q_{e}^{*}\nu \theta \left(\eta \right)}{\left(n+1\right)\kappa a{\left(x_{1}^{*}+b\right)}^{\frac{\left(n+1\right)}{2}}}$
Brownian diffusion parameter, $Nb=\frac{\rho c_{p}D_{B}\left(C_{s}^{*}-C_{\infty }^{*}\right)}{\rho_{f}C_{p}\nu }$	Thermophoresis diffusion parameter, $Nt=\frac{\rho C_{p}D_{T}\left(T_{s}^{*}-T_{\infty }^{*}\right)}{\rho_{f}C_{p}\nu T_{\infty }^{*}}$
Chemical reaction parameter, $K=\frac{2K_{r}}{\left(n+1\right)a{\left(x_{1}^{*}+b\right)}^{n-1}}$	Lewis number, $Le=\frac{\nu }{D_{B}}$
Bioconvection Lewis number, $Lb=\frac{\nu }{D_{m}}$	Peclet number, $Pe=\frac{bW_{c}}{D_{m}}$
Diffusion parameter, $L=\frac{RD_{B}\left(C_{s}^{*}-C_{\infty }^{*}\right)}{\kappa }$	Bioconvection diffusion parameter, $L^{*}=\frac{RD_{B}\left(N_{s}^{*}-N_{\infty }^{*}\right)}{\kappa }$
Concentration difference parameter, $\delta_{1}=\frac{C_{s}^{*}-C_{\infty }^{*}}{C_{\infty }^{*}}$	Microorganism concentration difference parameter, $\delta_{N}=\frac{N_{\infty }^{*}}{N_{s}^{*}-N_{\infty }^{*}}$

**Table 2:** Thermophysical features of nanofluid ([29,54]).

Properties	Mathematical Expression for Nanofluid
Viscosity	$\mu_{nf}=\frac{\mu_{f}}{{\left(1-\phi_{1}\right)}^{2.5}}$
Density	$\rho_{nf}=\left(1-\phi_{1}\right)\rho_{f}+\phi_{1}\rho_{s_{1}}$
Heat Capacity	${\left(\rho C_{p}\right)}_{nf}=\left(1-\phi_{1}\right){\left(\rho C_{p}\right)}_{f}+\phi_{1}{\left(\rho C_{p}\right)}_{s_{1}}$
Thermal Conductivity	$\frac{k_{nf}}{k_{f}}=\frac{k_{s_{1}}+\left(m-1\right)k_{f}-\left(m-1\right)\phi_{1}\left(k_{f}-k_{s_{1}}\right)}{k_{s_{1}}+\left(m-1\right)k_{f}+\phi_{1}\left(k_{f}-k_{s_{1}}\right)}$
Electrical Conductivity	$\frac{\sigma_{nf}}{\sigma_{f}}=\frac{\sigma_{s_{1}}+\left(m-1\right)\sigma_{f}-\left(m-1\right)\phi_{1}\left(\sigma_{f}-\sigma_{s_{1}}\right)}{\sigma_{s_{1}}+\left(m-1\right)\sigma_{f}+\phi_{1}\left(\sigma_{f}-\sigma_{s_{1}}\right)}$
Thermal Expansion Coefficient	${\left(\beta_{T}\right)}_{nf}=\left(1-\phi_{1}\right){\left(\beta_{T}\right)}_{f}+\phi_{1}{\left(\beta_{T}\right)}_{s_{1}}$
Concentration Thermal Expansion Coefficient	${\left(\beta_{C}\right)}_{nf}=\left(1-\phi_{1}\right){\left(\beta_{C}\right)}_{f}+\phi_{1}{\left(\beta_{C}\right)}_{s_{1}}$
Microorganism Thermal Expansion Coefficient	${\left(\beta_{N}\right)}_{nf}=\left(1-\phi_{1}\right){\left(\beta_{N}\right)}_{f}+\phi_{1}{\left(\beta_{N}\right)}_{s_{1}}$

**Table 3:** Thermophysical properties of nanoparticles and basefluids ([12,53]).

Physical Properties	Copper	PVA	Water
Density [ρ (kg/m${}^{3}$)]	8933	1020	997
Thermal Conductivity [κ (W/mK)]	400	0.2	0.613
Electrical Conductivity [σ (S/m)]	5.96 × 10^7^	11.7 × 10^−6^	0.05
Thermal Expansion Coefficient [$\beta_{T}\times$ 10${}^{-5}$ (K${}^{-1})$]	1.67	2.5	21
Specific Heat Capacity [$C_{p}$ (J/kgK)]	385	2000	4179

**Table 4:** Default Values of emerging parameters.

Parameters	Values	Parameters	Values	Parameters	Values	Parameters	Values
α	0.7	Pr	7.743	$\beta_{1}$	1	$\beta_{2}$	1
Ec	0.5	Nb=Nt	1	Le	0.3	*M*	0.1
Gr=Gc	0.3	Nc	0.1	*n*	0.5	$E_{1}$	0.1
*m*	0.9	$E_{t}$	1	*K*	0.5	φ	0.01
δ	1	$\delta_{1}$	0.5	$\delta_{N}$	1	Qe	0.3
Pe=Lb	0.3	*L*	1	$L^{*}$	1	$n_{1}$	3

## Data Availability

Not applicable.

## Nomenclature

Br	Brinkmann number	$Q_{e}$	Exponential heat source parameter
$T_{\infty }^{*}$	Ambient temperature (unit: K)	$Sh_{x}$	Sherwood number
$C_{\infty }^{*}$	Ambient concentration (unit: mol/m${}^{3}$ )	$T_{s}^{*}$	Temperature at the surface of the wall (unit: K)
$C_{f}$	Skin friction coefficient	$U_{s}$	Stretching sheet velocity (unit: m/s)
$C_{s}^{*}$	Concentration at the surface of the wall (unit: mol/m${}^{3}$ )	$u_{s}^{*}$	Velocity component in x-direction (unit: m/s)
$C_{p}$	Specific heat capacity (unit: mol Jkg${}^{-1}$ K${}^{-1}$ )	$v_{s}^{*}$	Velocity component in y-direction (unit: m/s)
$D_{B}$	Brownian diffusion coefficient (unit: kg m${}^{-1}$ s${}^{-1}$)	***Greek Letters***	
$D_{T}$	Thermophoresis diffusion coefficient (unit: kg m${}^{-1}$ s${}^{-1}$ K${}^{-1}$)	α	Thickness parameter
$E_{1}$	Electric field parameter	$\alpha_{1}$	Thermal diffusivity (unit: s/m${}^{2}$)
$E_{a}$	Dimensional activation energy (unit: KJ/mol )	$\beta_{C}$	Buoyancy force due to concentration (unit: K${}^{-1}$)
$E_{t}$	Nondimensional activation energy	$\beta_{T}$	Buoyancy force due to temperature (unit: K${}^{-1}$ )
Ec	Eckert number	$\beta_{1}$	Relaxation and retardation time ratio parameter
$G^{\prime }$	Dimensionless velocity	$\beta_{2}$	Retardation time parameter
Gr	Grashof number	ζ	Similarity variable
Gc	Solutal Grashof number	κ	Thermal conductivity (unit: W/(m.K))
$K_{r}$	Chemical reaction rate (unit: mol/s)	μ	Dynamic viscosity (unit: Pa.s)
*K*	Chemical reaction parameter	ν	Fluids’ kinematic viscosity (unit: m${}^{2}$ · s${}^{-1}$)
Le	Lewis number	$\lambda_{1}$	Relaxation and retardation time ratio
Lb	Bioconvection Lewis number	$L^{*}$	Microorganism diffusion parameter
*m*	Fitted rate constant	$\lambda_{2}$	Retardation time (unit: s)
*M*	Magnetic field parameter	ϕ	Dimensionless concentration (unit: mol/m${}^{3}$ )
Nt	Thermophoresis diffusion parameter	ρ	Density of fluid (unit: Kg/m${}^{3}$)
$Nu_{x}$	Nusselt number	ψ	Stream function (unit: Kg/(m.s))
Nb	Brownian diffusion parameter	σ	Electrical conductivity (unit: S/m )

## References

[1] Mushtaq A., Mustafa M., Hayat T., Alsaedi A. (2014). Nonlinear radiative heat transfer in the flow of nanofluid due to solar energy: A numerical study. J. Taiwan Inst. Chem. Eng..

[2] Shehzad S.A., Hayat T., Alsaedi A., Obid M.A. (2014). Nonlinear thermal radiation in three-dimensional flow of Jeffrey nanofluid: A model for solar energy. Appl. Math. Comput..

[3] Khan J.A., Mustafa M., Hayat T., Farooq M.A., Alsaedi A., Liao S. (2014). On model for three-dimensional flow of nanofluid: An application to solar energy. J. Mol. Liq..

[4] Khan J.A., Mustafa M., Hayat T., Alsaedi A. (2015). Three-dimensional flow of nanofluid over a non-linearly stretching sheet: An application to solar energy. Int. J. Heat Mass Transf..

[5] Zin N.A.M., Khan I., Shafie S. (2016). The impact silver nanoparticles on MHD free convection flow of Jeffrey fluid over an oscillating vertical plate embedded in a porous medium. J. Mol. Liq..

[6] Reddy K., Kamnapure N.R., Srivastava S. (2017). Nanofluid and nanocomposite applications in solar energy conversion systems for performance enhancement: A review. Int. J. Low-Carbon Technol..

[7] Daniel Y.S., Aziz Z.A., Ismail Z., Salah F. (2018). Impact of thermal radiation on electrical MHD flow of nanofluid over nonlinear stretching sheet with variable thickness. Alex. Eng. J..

[8] Wahab A., Hassan A., Qasim M.A., Ali H.M., Babar H., Sajid M.U. (2019). Solar energy systems–potential of nanofluids. J. Mol. Liq..

[9] Azam M., Shakoor A., Rasool H., Khan M. (2019). Numerical simulation for solar energy aspects on unsteady convective flow of MHD Cross nanofluid: A revised approach. Int. J. Heat Mass Transf..

[10] Sunitha G. (2019). Influence of thermal radiation on peristaltic blood flow of a Jeffrey fluid with double diffusion in the presence of gold nanoparticles. Informatics Med. Unlocked.

[11] Acharya N. (2020). On the flow patterns and thermal behaviour of hybrid nanofluid flow inside a microchannel in presence of radiative solar energy. J. Therm. Anal. Calorim..

[12] Song Y.Q., Obideyi B., Shah N.A., Animasaun I., Mahrous Y., Chung J.D. (2021). Significance of haphazard motion and thermal migration of alumina and copper nanoparticles across the dynamics of water and ethylene glycol on a convectively heated surface. Case Stud. Therm. Eng..

[13] Jamshed W., Eid M.R., Safdar R., Pasha A.A., Mohamed Isa S.S.P., Adil M., Rehman Z., Weera W. (2022). Solar energy optimization in solar-HVAC using Sutterby hybrid nanofluid with Smoluchowski temperature conditions: A solar thermal application. Sci. Rep..

[14] Sharma B.K., Poonam, Chamkha A.J. (2022). Effects of heat transfer, body acceleration and hybrid nanoparticles (Au-Al_2_O_3_) on MHD blood flow through a curved artery with stenosis and aneurysm using hematocrit-dependent viscosity. Waves Random Complex Media.

[15] Gandhi R., Sharma B., Kumawat C., Bég O.A. (2022). Modeling and analysis of magnetic hybrid nanoparticle (Au-Al_2_O_3_/blood) based drug delivery through a bell-shaped occluded artery with Joule heating, viscous dissipation and variable viscosity effects. Proc. Inst. Mech. Eng. Part E J. Process. Mech. Eng..

[16] Sharma B., Kumawat C., Vafai K. (2022). Computational biomedical simulations of hybrid nanoparticles (Au-Al_2_O_3_/blood-mediated) transport in a stenosed and aneurysmal curved artery with heat and mass transfer: Hematocrit dependent viscosity approach. Chem. Phys. Lett..

[17] Bhatti M., Ellahi R., Doranehgard M.H. (2022). Numerical study on the hybrid nanofluid (Co_3_O_4_-Go/H_2_O) flow over a circular elastic surface with non-Darcy medium: Application in solar energy. J. Mol. Liq..

[18] Bouslimi J., Alkathiri A.A., Althagafi T.M., Jamshed W., Eid M.R. (2022). Thermal properties, flow and comparison between Cu and Ag nanoparticles suspended in sodium alginate as Sutterby nanofluids in solar collector. Case Stud. Therm. Eng..

[19] Shahzad F., Jamshed W., Eid M.R., Safdar R., Putri Mohamed Isa S.S., El Din S.M., Mohd Nasir N.A.A., Iqbal A. (2022). Thermal cooling efficacy of a solar water pump using Oldroyd-B (aluminum alloy-titanium alloy/engine oil) hybrid nanofluid by applying new version for the model of Buongiorno. Sci. Rep..

[20] Pakravan H.A., Yaghoubi M. (2011). Combined thermophoresis, Brownian motion and Dufour effects on natural convection of nanofluids. Int. J. Therm. Sci..

[21] Anbuchezhian N., Srinivasan K., Chandrasekaran K., Kandasamy R. (2012). Thermophoresis and Brownian motion effects on boundary layer flow of nanofluid in presence of thermal stratification due to solar energy. Appl. Math. Mech..

[22] Kandasamy R., Muhaimin I., Mohamad R. (2013). Thermophoresis and Brownian motion effects on MHD boundary-layer flow of a nanofluid in the presence of thermal stratification due to solar radiation. Int. J. Mech. Sci..

[23] Hayat T., Asad S., Alsaedi A. (2015). Analysis for flow of Jeffrey fluid with nanoparticles. Chin. Phys. B.

[24] Mabood F., Ibrahim S., Khan W. (2016). Framing the features of Brownian motion and thermophoresis on radiative nanofluid flow past a rotating stretching sheet with magnetohydrodynamics. Results Phys..

[25] Sulochana C., Ashwinkumar G., Sandeep N. (2016). Transpiration effect on stagnation-point flow of a Carreau nanofluid in the presence of thermophoresis and Brownian motion. Alex. Eng. J..

[26] Astanina M., Abu-Nada E., Sheremet M. (2018). Combined effects of thermophoresis, brownian motion, and nanofluid variable properties on CuO-water nanofluid natural convection in a partially heated square cavity. J. Heat Transf..

[27] Awan S.E., Raja M.A.Z., Mehmood A., Niazi S.A., Siddiqa S. (2020). Numerical treatments to analyze the nonlinear radiative heat transfer in MHD nanofluid flow with solar energy. Arab. J. Sci. Eng..

[28] Rekha M., Sarris I.E., Madhukesh J., Raghunatha K., Prasannakumara B. (2022). Impact of thermophoretic particle deposition on heat transfer and nanofluid flow through different geometries: An application to solar energy. Chin. J. Phys..

[29] Sharma B., Kumar A., Gandhi R., Bhatti M. (2022). Exponential space and thermal-dependent heat source effects on electro-magneto-hydrodynamic Jeffrey fluid flow over a vertical stretching surface. Int. J. Mod. Phys. B.

[30] Sharma B., Khanduri U., Mishra N.K., Mekheimer K.S. (2022). Combined effect of thermophoresis and Brownian motion on MHD mixed convective flow over an inclined stretching surface with radiation and chemical reaction. Int. J. Mod. Phys. B.

[31] Sharma B., Gandhi R., Mishra N.K., Al-Mdallal Q.M. (2022). Entropy generation minimization of higher-order endothermic/exothermic chemical reaction with activation energy on MHD mixed convective flow over a stretching surface. Sci. Rep..

[32] Parvin S., Nasrin R., Alim M. (2014). Heat transfer and entropy generation through nanofluid filled direct absorption solar collector. Int. J. Heat Mass Transf..

[33] Khan M.I., Hayat T., Khan M.I., Waqas M., Alsaedi A. (2019). Numerical simulation of hydromagnetic mixed convective radiative slip flow with variable fluid properties: A mathematical model for entropy generation. J. Phys. Chem. Solids.

[34] Wang W.W., Cai Y., Wang L., Liu C.W., Zhao F.Y., Sheremet M.A., Liu D. (2020). A two-phase closed thermosyphon operated with nanofluids for solar energy collectors: Thermodynamic modeling and entropy generation analysis. Sol. Energy.

[35] Naz R., Noor M., Shah Z., Sohail M., Kumam P., Thounthong P. (2020). Entropy generation optimization in MHD pseudoplastic fluid comprising motile microorganisms with stratification effect. Alex. Eng. J..

[36] Farooq U., Munir S., Malik F., Ahmad B., Lu D. (2020). Aspects of entropy generation for the non-similar three-dimensional bioconvection flow of nanofluids. AIP Adv..

[37] Yusuf T.A., Mabood F., Prasannakumara B., Sarris I.E. (2021). Magneto-bioconvection flow of Williamson nanofluid over an inclined plate with gyrotactic microorganisms and entropy generation. Fluids.

[38] Li Y.X., Khan M.I., Gowda R.P., Ali A., Farooq S., Chu Y.M., Khan S.U. (2021). Dynamics of aluminum oxide and copper hybrid nanofluid in nonlinear mixed Marangoni convective flow with entropy generation: Applications to renewable energy. Chin. J. Phys..

[39] Sharma B., Gandhi R., Bhatti M. (2022). Entropy analysis of thermally radiating MHD slip flow of hybrid nanoparticles (Au-Al_2_O_3_/Blood) through a tapered multi-stenosed artery. Chem. Phys. Lett..

[40] Khanduri U., Sharma B.K. (2022). Entropy Analysis for MHD Flow Subject to Temperature-Dependent Viscosity and Thermal Conductivity. Nonlinear Dynamics and Applications.

[41] Salawu S., Obalalu A., Shamshuddin M. (2022). Nonlinear solar thermal radiation efficiency and energy optimization for magnetized hybrid Prandtl–Eyring nanoliquid in aircraft. Arab. J. Sci. Eng..

[42] Avramenko A., Kuznetsov A. (2004). Stability of a suspension of gyrotactic microorganisms in superimposed fluid and porous layers. Int. Commun. Heat Mass Transf..

[43] Avramenko A., Kuznetsov A. (2010). The onset of bio-thermal convection in a suspension of gyrotactic microorganisms in a fluid layer with an inclined temperature gradient. Int. J. Numer. Methods Heat Fluid Flow.

[44] Mutuku W.N., Makinde O.D. (2014). Hydromagnetic bioconvection of nanofluid over a permeable vertical plate due to gyrotactic microorganisms. Comput. Fluids.

[45] Acharya N., Das K., Kundu P.K. (2016). Framing the effects of solar radiation on magneto-hydrodynamics bioconvection nanofluid flow in presence of gyrotactic microorganisms. J. Mol. Liq..

[46] Saleem S., Rafiq H., Al-Qahtani A., El-Aziz M.A., Malik M., Animasaun I. (2019). Magneto Jeffrey nanofluid bioconvection over a rotating vertical cone due to gyrotactic microorganism. Math. Probl. Eng..

[47] Sohail M., Naz R., Abdelsalam S.I. (2020). On the onset of entropy generation for a nanofluid with thermal radiation and gyrotactic microorganisms through 3D flows. Phys. Scr..

[48] Song Y.Q., Hamid A., Khan M.I., Gowda R.P., Kumar R.N., Prasannakumara B., Khan S.U., Khan M.I., Malik M. (2021). Solar energy aspects of gyrotactic mixed bioconvection flow of nanofluid past a vertical thin moving needle influenced by variable Prandtl number. Chaos Solitons Fractals.

[49] Naidu K.K., Babu D.H., Reddy S.H., Narayana P.S. (2021). Radiation and partial slip effects on magnetohydrodynamic Jeffrey nanofluid containing gyrotactic microorganisms over a stretching surface. J. Therm. Sci. Eng. Appl..

[50] Sharma B.K., Khanduri U., Mishra N.K., Chamkha A.J. (2022). Analysis of Arrhenius activation energy on magnetohydrodynamic gyrotactic microorganism flow through porous medium over an inclined stretching sheet with thermophoresis and Brownian motion. Proc. Inst. Mech. Eng. Part E J. Process. Mech. Eng..

[51] Bhatti M., Arain M., Zeeshan A., Ellahi R., Doranehgard M. (2022). Swimming of Gyrotactic Microorganism in MHD Williamson nanofluid flow between rotating circular plates embedded in porous medium: Application of thermal energy storage. J. Energy Storage.

[52] Hussain A., Farooq N. (2023). Gyrotactic micro-organisms swimming under the Hyperbolic Tangent Blood Nano Material and Solar biomimetic system over the Esophagus. Int. Commun. Heat Mass Transf..

[53] Shahzad F., Jamshed W., Nisar K.S., Khashan M.M., Abdel-Aty A.H. (2021). Computational analysis of Ohmic and viscous dissipation effects on MHD heat transfer flow of Cu-PVA Jeffrey nanofluid through a stretchable surface. Case Stud. Therm. Eng..

[54] Ali A., Maqsood M., Anjum H., Awais M., Sulaiman M. (2022). Analysis of heat transfer on MHD Jeffrey nanofluid flow over nonlinear elongating surface of variable thickness. ZAMM-J. Appl. Math. Mech. Angew. Math. Mech..

[55] Muhammad T., Waqas H., Manzoor U., Farooq U., Rizvi Z.F. (2022). On doubly stratified bioconvective transport of Jeffrey nanofluid with gyrotactic motile microorganisms. Alex. Eng. J..

